# Tryptophan Metabolism Acts as a New Anti‐Ferroptotic Pathway to Mediate Tumor Growth

**DOI:** 10.1002/advs.202204006

**Published:** 2023-01-10

**Authors:** Dong Liu, Chun‐hui Liang, Bin Huang, Xiao Zhuang, Weiwei Cui, Li Yang, Yinghong Yang, Yudan Zhang, Xiaolong Fu, Xiaoju Zhang, Lutao Du, Wei Gu, Xiangdong Wang, Chengqian Yin, Renjie Chai, Bo Chu

**Affiliations:** ^1^ Department of Cell Biology School of Basic Medical Sciences Cheeloo College of Medicine Shandong University Jinan Shandong 250012 China; ^2^ Institute for Cancer Research Shenzhen Bay Laboratory Shenzhen 518107 China; ^3^ Department of Respiratory and Critical Care Medicine Zhengzhou University People's Hospital Henan Provincial People's Hospital Zhengzhou University Zhengzhou Henan 450000 China; ^4^ State Key Laboratory of Bioelectronics Department of Otolaryngology Head and Neck Surgery Zhongda Hospital School of Life Sciences and Technology Advanced Institute for Life and Health Jiangsu Province High‐Tech Key Laboratory for Bio‐Medical Research Southeast University Nanjing 210096 China; ^5^ Department of Clinical Laboratory The Second Hospital of Shandong University Jinan Shandong 250033 China; ^6^ Institute for Cancer Genetics and Department of Pathology and Cell Biology Herbert Irving Comprehensive Cancer Center College of Physicians and Surgeons Columbia University 1130 Nicholas Ave New York NY 10032 USA; ^7^ Co‐Innovation Center of Neuroregeneration Nantong University Nantong 226001 China; ^8^ Department of Otolaryngology Head and Neck Surgery, Sichuan Provincial People's Hospital University of Electronic Science and Technology of China Chengdu 610000 China

**Keywords:** 3‐HA, 5‐HT, ferroptosis, 3‐hydroxyanthranilate 3,4‐dioxygenase (HAAO), kynureninase (KYNU, monoamine oxidase A (MAOA), tryptophan

## Abstract

Emerging evidence reveals that amino acid metabolism plays an important role in ferroptotic cell death. The conversion of methionine to cysteine is well known to protect tumour cells from ferroptosis upon cysteine starvation through transamination. However, whether amino acids‐produced metabolites participate in ferroptosis independent of the cysteine pathway is largely unknown. Here, the authors show that the tryptophan metabolites serotonin (5‐HT) and 3‐hydroxyanthranilic acid (3‐HA) remarkably facilitate tumour cells to escape from ferroptosis distinct from cysteine‐mediated ferroptosis inhibition. Mechanistically, both 5‐HT and 3‐HA act as potent radical trapping antioxidants (RTA) to eliminate lipid peroxidation, thereby inhibiting ferroptotic cell death. Monoamine oxidase A (MAOA) markedly abrogates the protective effect of 5‐HT via degrading 5‐HT. Deficiency of MAOA renders cancer cells resistant to ferroptosis upon 5‐HT treatment. Kynureninase (KYNU), which is essential for 3‐HA production, confers cells resistant to ferroptotic cell death, whereas 3‐hydroxyanthranilate 3,4‐dioxygenase (HAAO) significantly blocks 3‐HA mediated ferroptosis inhibition by consuming 3‐HA. In addition, the expression level of HAAO is positively correlated with lipid peroxidation and clinical outcome. Together, the findings demonstrate that tryptophan metabolism works as a new anti‐ferroptotic pathway to promote tumour growth, and targeting this pathway will be a promising therapeutic approach for cancer treatment.

## Graphical abstract

Tryptophan metabolites 5‐HT and 3‐HA act as potent radical trapping antioxidants to inhibit ferroptosis both in vitro and in vivo. Tryptophan metabolites‐mediated ferroptosis suppression is a new anti‐ferroptosis pathway distinct from cysteine‐mediated ferroptosis inhibition or any other classical anti‐ferroptotic pathways to facilitate tumor development. Therefore, targeting this pathway will be a promising therapeutic approach for cancer treatment.

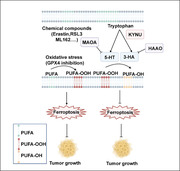

## 1 Introduction

Ferroptosis is a newly identified programmed cell death that is distinct from apoptosis and necrosis.^[^ ^1^ ^]^ The initiation of ferroptotic cell death is induced by accumulated peroxidized phospholipids in an iron‐dependent manner.^[^ ^1^, ^2^ ^]^ The lipid hydroperoxides (PUFA‐OOH) are converted into non‐toxic lipid alcohols (PUFA‐OH) by glutathione peroxidase 4 (GPX4), ferroptosis suppressor protein 1(FSP1), tetrahydrobiopterin (BH4) system or newly identified dihydroorotate dehydrogenase (DHODH).^[^ ^2^, ^3^ ^]^ Emerging evidence indicates that ferroptosis is implicated in cancer immunotherapy and tumor suppression.^[^ ^4^ ^]^ Among these four identified anti‐ferroptosis pathway, GPX4‐GSH pathway occupies the dominant position to defend ferroptosis for cancer cells.

Cysteine has been shown to suppress ferroptosis via biosynthesis of glutathione (GSH), which is a substrate for GPX4 to diminish oxidized lipids.^[^ ^2a^ ^]^ The source of cysteine mainly depends on extracellular uptake and de novo biosynthesis. Solute carrier family 7 member 11 (SLC7A11), the catalytic subunit of the cystine/glutamate antiporter system x_c_^−^ (SLC7A11/SLC3A2), is the major plasma membrane transporter of extracellular cystine.^[^ ^5^ ^]^ Intracellular cystine is rapidly reduced to cysteine, which is subsequently utilized for glutathione synthesis. GPX4 eliminates lipid hydroperoxides by utilizing GSH to protect cells against membrane lipid peroxidation. Therefore, blocking SLC7A11‐mediated cystine uptake via cystine starvation or erastin treatment sensitizes cells to ferroptosis.^[^ ^1^ ^]^ Under cystine starvation or cystine‐deficient condition, some tumor cells could defend ferroptosis by conversion of methionine to cysteine through transamination pathway.^[^ ^6^ ^]^ Increasing studies revealed some other amino acids such as glutamate and glutamine participate in ferroptosis via cysteine‐related pathway.^[^ ^1^, ^7^ ^]^ However, little is known whether the metabolites derived from amino acids affect ferroptotic sensitivity of tumor cells independent of cysteine‐mediated ferroptosis.

Here we demonstrate the role of tryptophan metabolism pathway‐mediated ferroptosis resistance. Our work shows that the tryptophan metabolites 5‐HT and 3‐HA significantly enhance the anti‐ferroptosis activity of tumor cells in a relatively low concentration. Mechanistically, both 5‐HT and 3‐HA remarkably eliminate oxidized phospholipids as potent radical trapping antioxidants (RTA). By investigation of the enzymes involved in 5‐HT and 3‐HA metabolism, we find that MAOA remarkably abrogates the protective effect of 5‐HT via degrading 5‐HT. Loss of MAOA efficiently rescues 5‐HT mediated ferroptosis inhibition. KYNU confers tumor cells resistant to ferroptosis by generation of 3‐HA, whereas deficiency of KYNU significantly enhances ferroptotic sensitivity. In contrast, HAAO markedly abolishes 3‐HA mediated ferroptosis inhibition. Additionally, the expression level of HAAO is positively correlated with lipid peroxidation and improved overall survival in the patients with multiple types of cancers. Together, our findings reveal that tryptophan metabolism works as a new pathway for tumor cells to escape from ferroptosis, and targeting this pathway will be beneficial for cancer therapy.

## 2 Results

### 2.1 Identification and Validation of Tryptophan Metabolites as Endogenous Potent Ferroptosis Suppressors

**Figure 1:**
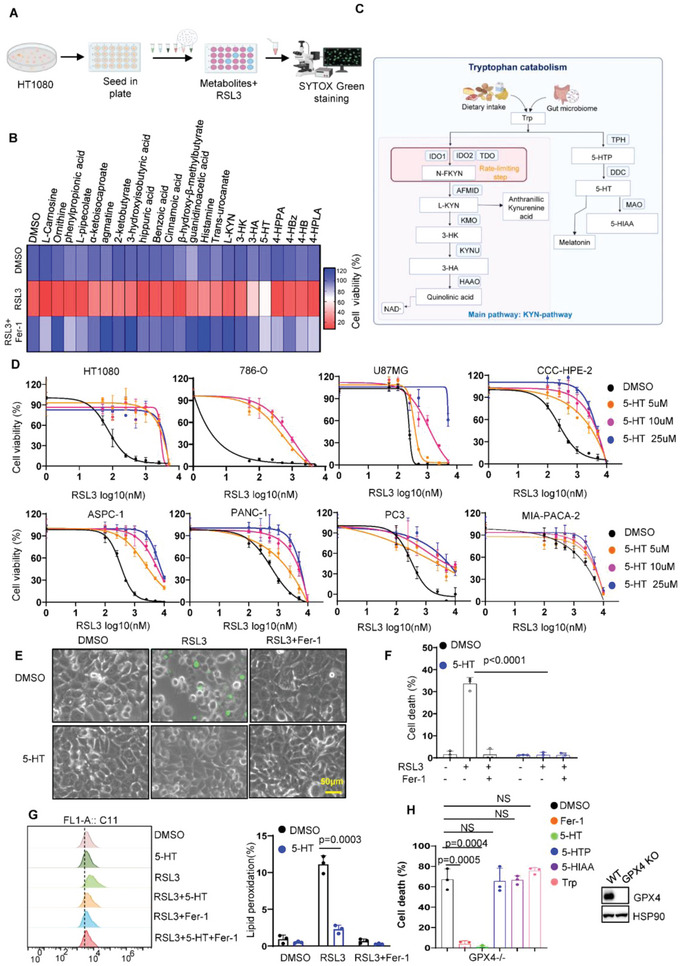
Identification and validation of tryptophan metabolites as potent ferroptosis inhibitor. A) Schematic of the identification of potential ferroptosis‐resistant amino acid‐derived metabolites, using HT1080 cells pretreated with indicated metabolites followed by RSL3 treatment for 24 h. B) Heatmap data showing the change of ferroptotic cell death upon amino acid metabolites treatment in HT1080 cells. C) Schematic diagram of tryptophan metabolism pathway. Trp, L‐tryptophan; N‐FKYN, *N*‐formyl‐kynurenine; L‐KYN, L‐kynurenine; 3‐HK, 3‐hydroxykynurenine; 3‐HA, 3‐hydroxyanthranillic acid; 5‐HTP, 5‐hydroxytryptophan; 5‐HT, serotonin; 5‐HIAA, 5‐hydroxyindoleacetic acid. D) Dose‐dependent toxicity of RSL3 induced cell death in a panel of cancer cell lines with or without 5‐HT treatment. Cell viability was assessed 24 h thereafter using CCK8. E) Representative phase‐contrast images of HT1080 cells treated with 5‐HT (10 µm), RSL3 (200 nm), and Fer‐1 (1 µm) for 8 h. Dead cells were stained with Sytox Green. Scale bars, 50 µm. F) Cell death measurement of HT1080 cells treated with 5‐HT (10 µm), RSL3 (200 nm), and Fer‐1 (1 µm) for 8 h. G) Lipid peroxidation measurements in HT1080 cells treated with 5‐HT (10 µm), RSL3 (200 nm), and Fer‐1 (1 µm) for 4 h. H) Cell death measurement of HT1080 GPX4^‐/‐^ cells treated with tryptophan (10 µm), 5‐HTP (10 µm), 5‐HT (10 µm), 5‐HIAA (10 µm), RSL3 (200 nm), and Fer‐1 (1 µm) for 8 h.

To systematically test whether amino acid metabolism impacts ferroptotic sensitivity of cancer cells independent of cysteine pathway, we performed a metabolic library screening containing 23‐ amino acid metabolites (final concentration at 5 µm) for ferroptosis assay in the ferroptosis‐susceptible fibrosarcoma‐derived HT1080 cells (**Figure** **1**A). In this screen, we excluded the metabolites that are substrates for TCA cycle and cysteine biosynthesis, since mitochondria and cysteine‐involved ferroptosis was well described.^[^ ^1^, ^2^, ^7^, ^8^ ^]^ Strikingly, only metabolites derived from tryptophan metabolism 5‐HT and 3‐HA substantially protected tumor cells against RSL3‐induced cell death, whereas other amino acid metabolites displayed no or limited effects (Figure 1B, Figures S1A–F,S2A,B,S3A, Supporting Information). A recent study revealed that metabolic intermediates generated from tyrosine were required for CoQ10 synthesis,^[^ ^9^ ^]^ and it was also reported that tyrosine‐derived 4‐hydroxyphenylpyruvate (4‐HPPA) at a high concentration inhibits ferroptosis at least partially via NRF2 pathway,^[^ ^10^ ^]^ we therefore wondered whether tyrosine derivates are involved in suppression of RSL3‐induced ferroptosis (Figure S3B, Supporting Information). However, none of tyrosine metabolites including tyrosine, 4‐HPPA, 4‐HPLA, 4‐HB, and 4‐HBz had the ability to protect cells from ferroptosis at a concentration of up to 50 µm (Figure 1B; Figure S3C, Supporting Information). Interestingly, analysis of tryptophan metabolism revealed that 5‐HT is involved in the pathway of serotonin synthesis, while 3‐HA is generated from kynurenine pathway (Figure 1C), we therefore focused on the two major tryptophan pathways, respectively.

We first explored the role of 5‐HT in ferroptosis resistance. As shown in Figure 1D, 5‐HT substantially rendered a panel of cancer cell lines resistant to RSL3 induced ferroptosis. In addition, 5‐HT completely blocked RSL3 induced cell death indicated by the dye SYTOX Green (Figure 1E,F). Since lipid peroxidation is a hallmark of ferroptosis, we next estimated the levels of lipid peroxidation in 5‐HT‐incubated HT1080 cells by staining of BODIPY‐C11 581/591). As expected, 5‐HT effectively eliminated lipid peroxidation induced by RSL3 (Figure 1G). Four major metabolites 5‐hydroxytryptophan (5‐HTP), 5‐HT, melatonin, and 5‐hyroxyindole acetic acid (5‐HIAA) are produced in serotonin pathway, in which melatonin has been found to suppress ferroptosis via activation of NRF2 signaling pathway.^[^ ^11^ ^]^ Surprisingly, we found that 5‐HT exhibited stronger protection than melatonin (Figure S4A, Supporting Information). Moreover, supplementation of 5‐HT has no effect on NRF2 pathway (Figure S4B, Supporting Information) and completely abolishes ferroptosis in NRF2 KO cell lines (Figure S4C, Supporting Information), indicating that 5‐HT‐mediated ferroptosis is independent of NRF2. Although 5‐HTP and 5‐HIAA have similar chemical structure with 5‐HT, the screen data revealed 5‐HTP and 5‐HIAA has no or weak capacity to protect cells from ferroptosis upon RSL3 treatment or GPX4 inactivation (Figure 1H; Figure S4D–F, Supporting Information), suggesting that 5‐HT is a potent ferroptosis suppressor in serotonin pathway.

### 2.2 5‐HT Mediated Ferroptosis Inhibition is Independent of 5‐HT Receptors or Canonical Ferroptosis Pathways

**Figure 2:**
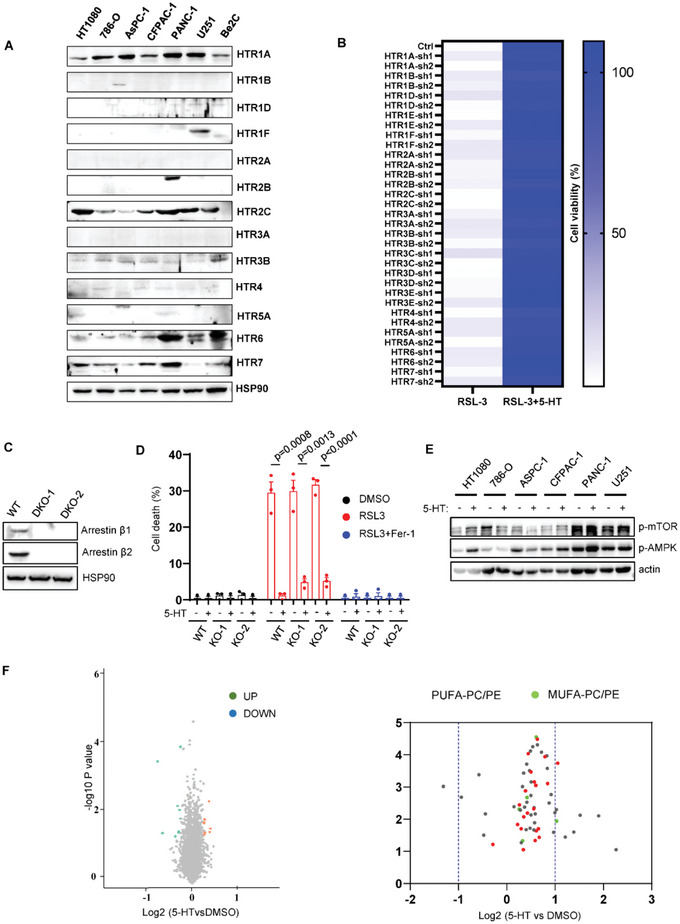
5‐HT mediated ferroptosis resistance is independent of canonical anti‐ferroptosis pathway. A) Western blot analysis of 5‐HT receptors in a panel of cancer cell lines. B) HT1080 cells expressing shRNA of 5‐HT receptors were treated with RSL3 (200 nm) and 5‐HT (10 µm). Cell viability was assessed 24 h thereafter using CCK8. C) Western blot analysis of arrestin *β*1 and arrestin *β*2 in 293T arrestin *β*1^‐/‐^, arrestin *β*2^‐/‐^ DKO cells. D) Cell death measurement of 293T arrestin *β*1^‐/‐^, arrestin *β*2^‐/‐^ DKO cells treated with RSL3 (2.5 µm) and Fer‐1 (1 µm) for 8 h. E) Western blot analysis of p‐mTOR and p‐AMPK in a panel of cell lines upon 5‐HT treatment (10 µm) for 12 h. F) RNA‐seq analysis of HT1080 cells with or without 5‐HT treatment (10 µm) for 12 h. G) Volcano plots showing the alteration of phospholipid components in HT1080 cells treated with 5‐HT (10 µm) for 12 h.

5‐HT is well known as the ligand of 5‐HT receptors (HTRs) to activate the downstream cellular signaling pathway.^[^ ^12^ ^]^ HTRs containing 17 members can be divided into seven sub‐families: HTR1, HTR2, HTR3, HTR4, HTR5, HTR6, and HTR7. To understand whether 5‐HT renders cells resistant to ferroptosis via HTRs, we first examined the expression level of HTRs in a panel of cancer cell lines. As shown in **Figure** **2**A, most of HTR isoforms are weakly detected or undetectable. We further performed shRNA‐targeted HTRs screen to test whether HTRs are essential for 5‐HT‐mediated ferroptosis suppression. The cell viability assay showed knockdown of any isoform of HTRs was unable to restore RSL3 induced ferroptosis upon 5‐HT treatment (Figure 2B). HTRs, which belong to G‐protein coupled receptor (GPCR), could modulate activation of alternative down‐stream signaling pathway via beta‐arrestin (*β*‐arrestin) family members.^[^ ^13^ ^]^ We therefore established *β*‐arrestin single or double knockout (DKO) clones in 293T cells using CRISPR‐Cas9 technology to test whether loss of beta‐arrestin diminished the protective effect of 5‐HT. As shown in Figure 2C and Figure S5A,B, Supporting Information, arrestin‐1 and 2 were undetectable in single or DKO cells. However, we did not observe obvious change 5‐HT mediated ferroptosis resistance (Figure 2D; Figure S5A,B, Supporting Information). Furthermore, GPCR signaling Gs inhibitor (NF449) or Gi inhibitor (PTX) has little effect to restore ferroptotic cell death (Figure S5C, Supporting Information), indicating that HTRs‐mediated cellular signaling are dispensable for the protective phenotype of 5‐HT.

Previous studies showed that 5‐HT could modulate gene expression through histone serotonylation,^[^ ^14^ ^]^ we supposed that 5‐HT might regulate ferroptosis through modification of classical ferroptosis‐related genes. Supplementation with 5‐HT did not affect the status of a variety of ferroptosis‐related genes including GPX4, FSP1, DHODH, GCH1, SLC7A11, and ACSL4 (Figure S5D, Supporting Information). To further validate this finding, we generated KO clones for these genes in cancer cells (Figure S5E–H, Supporting Information). We observed none of these CRISPR KO clones abrogated 5‐HT mediated anti‐ferroptotic effect (Figure S5E–H, Supporting Information), suggesting that 5‐HT‐regulated ferroptosis inhibition is independent of classical ferroptosis pathway. In addition, 5‐HT has been found to regulate the status of mTOR and AMPK pathways which are recently identified to regulate ferroptosis.^[^ ^15^ ^]^ However, 5‐HT treatment did not induce significant alteration of p‐mTOR or p‐AMPK except in HT1080 cells (Figure 2E). Additionally, we performed proteomics experiments in HT1080 supplemented with 5‐HT and found no obvious change in protein expression (Figure 2F). These data indicate that 5‐HT‐mediated ferroptosis is distinct from conventional anti‐ferroptosis pathways.

As fatty acid composition is the major cause of lipid peroxidation,^[^ ^2b^ ^]^ we performed non‐targeted lipidomics assay to test whether 5‐HT affects the composition of fatty acid content. As shown in Figure 2G, 5‐HT supplementation does not significantly interfere with fatty acid pathway. Taken together, these findings indicate that 5‐HT renders cells resistant to ferroptosis independent of canonical ferroptosis mechanisms or HTRs.

### 2.3 5‐HT Acts as RTA to Suppress Ferroptotic Cell Death

**Figure 3:**
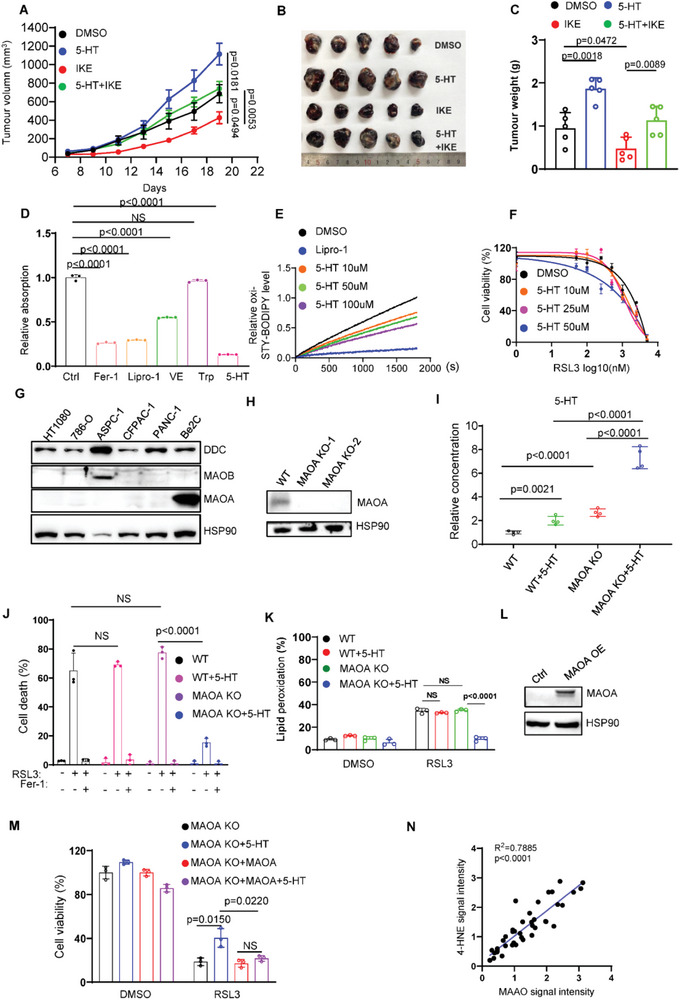
MAOA decreases the levels of cellular 5‐HT to suppress anti‐ferroptotic role of 5‐HT. A) Tumor volumes of B16F10 xenograft in C57BL/6 mice (*n* = 5) supplemented with 5‐HT (10 mg kg^−1^) or IKE (40 mg kg^−1^). B) Representative images of B16F10 xenograft in C57BL/6 mice (*n* = 5) supplemented with 5‐HT (10 mg kg^−1^) or IKE (40 mg kg^−1^). C) Tumor weight of B16F10 xenograft in C57BL/6 mice (*n* = 5) supplemented with 5‐HT (10 mg kg^−1^) or IKE (40 mg kg^−1^). D) DPPH assay to test the ability of 5‐HT (10 µm) as radical trapping agent, fer‐1 (10 µm), lipro‐1 (10 µm), and Vitamin E (10 µm) as positive controls. E) Representative autoxidation of STY‐BODIPY (1 µm)‐embedded liposomes of egg phosphatidylcholine lipids (1 mm, ≈100‐nm particle size) suspended in PBS, initiated by 0.2 mm DTUN containing lipro‐1 (2.5 µm) or 5‐HT for the indicated concentrations. F) Dose‐dependent toxicity of RSL3 induced cell death in Be2C cells with or without 5‐HT treatment. Cell viability was assessed 24 h thereafter using CCK8. G) Western blot analysis of DDC, MAOA, and MAOB levels in a panel of cancer cells lines. H) Western blot analysis showed that MAOA is undetectable in Be2C MAOA^‐/‐^ clones. I) LC‐MS based quantitative metabolomics of cellular 5‐HT in Be2C WT and MAOA KO cells with or without 5‐HT (10 µm) treatment. J) Cell death and K) lipid peroxidation measurement of Be2C WT and MAOA KO cells treated with RSL3 (5 µm) and 5‐HT (10 µm) for 12 h. L) Western blot analysis of MAOA in Be2C MAOA^‐/‐^ cells transfected with empty or MAOA vector. M) Cell viability of Be2C MAOA^‐/‐^ cells transfected with empty or MAOA vector treated with RSL3 (5 µm) for 12 h. N) Dot plot depicting the relevance of the signal intensity of MAOA and 4‐HNE IHC staining in the indicated kidney renal clear cell carcinoma samples.

To demonstrate whether 5‐HT contributes to tumor development in vivo, B16F10 xenograft tumors were treated with 5‐HT for 2 weeks. As shown in **Figure** **3**A–C, supplementation of 5‐HT significantly facilitates tumor development. Moreover, imidazole ketone erastin (IKE)‐induced tumor suppression was greatly inhibited by supplementation of 5‐HT, indicating that 5‐HT‐mediated ferroptosis inhibition contributes to tumor growth both in vitro and in vivo. Classical ferroptosis inhibitors such as ferrostatin‐1 (Fer‐1), liprostatin‐1 (Lipro‐1), and vitamin E (VE) eliminate free radicals. 5‐HT has been reported to diminish lipid peroxidation in the liposome oxidation assay in vitro.^[^ ^16^ ^]^ Therefore, we speculated whether 5‐HT inhibits ferroptosis via blockade of accumulation of peroxidized phospholipids as RTA. To explore the possibility that 5‐HT acts as RTA to inhibit ferroptosis, we first examined tryptophan metabolites activity to capture free radicals by DPPH assay. As expected, Fer‐1, Lipro‐1, and VE significantly decreased the levels of free radicals (Figure 3D). Interestingly, 5‐HT strongly eliminates free radical whereas tryptophan has no effect compared to control (Figure 3D). A newly identified fluorescence‐enabled inhibited autoxidation (FENIX) approach, which is more superior and accurate than DPPH, enables reliable prediction of anti‐ferroptotic activity of redox‐active compounds.^[^ ^17^ ^]^ We therefore performed FENIX assay and found that 5‐HT effectively blocked oxidation of liposomes in a dose‐dependent manner (Figure 3E), suggesting 5‐HT is a potent RTA to repress ferroptosis via decreasing the level of oxidized phospholipids.

### 2.4 MAOA‐Induced 5‐HT Consumption Sensitizes Cells to Ferroptosis

Among a variety of cell lines, Be2C cell line is the only one which does not respond to 5‐HT‐participated protection (Figure 3F). We wondered whether dysregulated enzymatic activity of serotonin pathway contributed to this phenotype in Be2C cells. To explore this hypothesis, we examined the expression levels of these enzymes (Figure 3G). MAOA, a rate‐limiting enzyme degrading 5‐HT, is dominantly expressed in Be2C cells, whereas MAOB was almost undetectable except in ASPC‐1 cells (Figure 3G). To investigate whether MAOA is the major factor to abrogate the protective effect of 5‐HT, we established MAOA KO Be2C cell line using CRISPR‐Cas9 technology (Figure 3H). As expected, loss of MAOA significantly increased the basal level of cellular 5‐HT (Figure 3I). Furthermore, supplementation of 5‐HT substantially raised the cellular abundance of 5‐HT in MAOA KO cells, whereas weak increase in WT cells. Knockout of MAOA restored the resistance to ferroptosis upon supplementation with 5‐HT (Figure 3J,K). Notably, ectopic expression of MAOA in MAOA KO cells abrogated 5‐HT‐mediated ferroptosis inhibition (Figure 3L,M). Furthermore, through the gene expression analysis of TIMER database (http://timer.comp‐genomics.org/), we found that MAOA expression is lower in multiple types of cancers compared to normal tissues (Figure S6A, Supporting Information). Moreover, Kaplan–Meier survival analysis demonstrated that high expression of MAOA is associated with improved overall survival in patients with various cancer types including breast cancer, kidney renal clear cell carcinoma, lung adenocarcinoma and bladder carcinoma (Figure S6B, Supporting Information). To further demonstrate whether MAOA is correlated to ferroptosis in cancer samples, we detected the correlation of MAOA with ferroptosis by immunohistochemistry (IHC) staining of MAOA and 4‐HNE in human kidney renal clear cell carcinoma samples. As shown in Figure 3N and Figure S6C, Supporting Information, IHC staining of MAOA was highly related to 4‐HNE staining in the human kidney cancer tissue microarray, indicating that MAOA is closely related to ferroptosis in human cancers. Taken together, these data demonstrate that MAOA inhibits tumor growth via suppressing the protective effect of 5‐HT, implying a potential role of MAOA as a tumor suppresser via ferroptosis.

### 2.5 KYNU Is Essential for 3‐HA Biosynthesis and Ferroptosis Resistance

**Figure 4:**
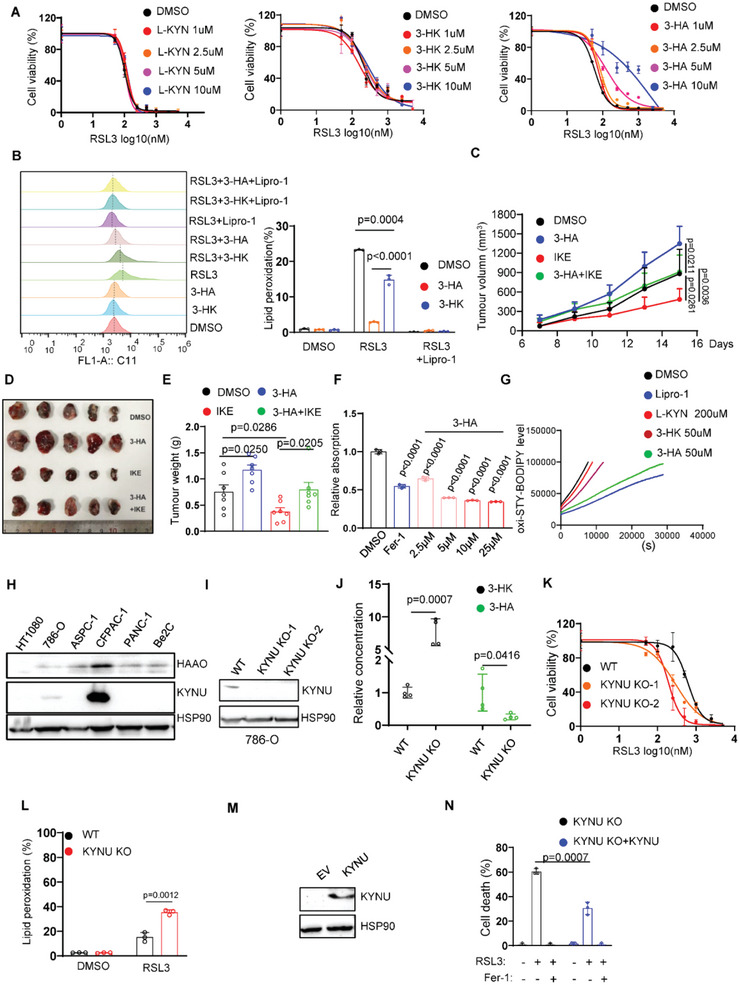
KYNU‐mediated 3‐HA biosynthesis efficiently inhibits ferroptotic cell death. A) Dose‐dependent toxicity of RSL3 induced cell death in HT1080 cells upon L‐KYN, 3‐HK, or 3‐HA treatment for the indicated concentrations. Cell viability was assessed 24 h thereafter using CCK8. B) Lipid peroxidation measurements in HT1080 cells treated with RSL3 (200 nm), 3‐HK (10 µm), 3‐HA (10 µm), and lipro‐1 (1 µm) for 4 h. C) Tumor volumes of B16F10 xenograft in C57BL/6 mice (*n* = 5) supplemented with 3‐HA (20 mg kg^−1^) or IKE (40 mg kg^−1^). D) Representative images of B16F10 xenograft in C57BL/6 mice (*n* = 5) supplemented with 3‐HA (20 mg kg^−1^) or IKE (40 mg kg^−1^). E) Tumor weight of B16F10 xenograft in C57BL/6 mice (*n* = 5) supplemented with 3‐HA (20 mg kg^−1^) or IKE (40 mg kg^−1^). F) DPPH assay to test the ability of 5‐HT for the indicated concentration as radical trapping agent, fer‐1 (10 µm) as positive controls. G) Representative autoxidation of STY‐BODIPY (1 µm)‐embedded liposomes of egg phosphatidylcholine lipids (1 mm, ≈100‐nm particle size) suspended in PBS, initiated by 0.2 mm DTUN containing lipro‐1 (2.5 µm) or tryptophan metabolites for the indicated concentration. H) Western blot analysis of KYNU levels in a panel of cancer cells lines. I) Western blot analysis of KYNU in 786‐O KYNU^‐/‐^ clones. J) LC‐MS based quantitative metabolomics of cellular 3‐HK and 3‐HA in 786‐O WT and KYNU^‐/‐^ cells. K) Cell viability and L) lipid peroxidation of 786‐O WT and KYNU^‐/‐^ cells treated with RSL3 for the indicated concentration. M) Western blot analysis of KYNU in 786‐O KYNU^‐/‐^ cells transfected with empty or KYNU vector. N) Cell death measurement of 786‐O KYNU^‐/‐^ cells transfected with empty or KYNU vector treated with RSL3 (250 nm) for 8 h.

We further explored the mechanism of kynurenine pathway in ferroptosis. 3‐HA, L‐kynurenine (L‐KYN), and 3‐hydroxy‐kynurenine (3‐HK) are the major metabolites involved in this pathway. Aforementioned data (Figure 1B) showed 3‐HA, not any other kynurenine metabolites, significantly protected cells from ferroptosis at a low concentration (5 µm). Supplementation with these metabolites with a range of concentration (1–10 µm) exhibited similar phenotype (**Figure** **4**A). Moreover, supplementation with 3‐HA in HT1080 cells remarkedly inhibited RSL3 induced lipid peroxidation, whereas 3‐HK exhibited a minor effect (Figure 4B). These findings indicate that 3‐HA is the most efficient ferroptosis suppressor in kynurenine pathway. To ascertain whether 3‐HA contributes to ferroptosis‐mediated tumor development in vivo, we tested whether administration of 3‐HA affects tumor cell growth with or without imidazole ketone erastin (IKE) treatment. As HT1080 and 786‐O have weak capacity for tumorigenesis in vivo (data not shown), B16F10 cell line, which responds to 3‐HA mediated ferroptosis inhibition (Figure S7A, Supporting Information), is used for xenograft assay. As expected, supplementation of 3‐HA significantly promoted tumor development (Figure 4C–E). In addition, IKE‐induced tumor suppression was markedly abrogated by 3‐HA. These data demonstrate that 3‐HA‐mediated ferroptosis inhibition contributes to tumor growth both in vitro and in vivo.

A most recent work revealed that L‐KYN (200 µm) and 3‐HK (25–200 µm) could render Hela cells resistant to erastin or RSL3 induced ferroptosis and this protective effect is partially dependent of NRF2‐SLC7A11 pathway.^[^ ^18^ ^]^ Consistent with this finding, 3‐HK and 3‐HA efficiently suppressed erastin or RSL3 induced ferroptosis in HT1080, 786‐O, and OVCAR8 cells. However, we did not observe the protective effect of L‐KYN (Figure S7B–D, Supporting Information). To confirm whether kynurenine‐derived metabolites inhibits ferroptosis via NRF2 pathway, we treated cells with these metabolites and detected the activation of NRF2 pathway. Indeed, all the tryptophan derivates activated NRF2 and its target genes SLC7A11 (Figure S8A, Supporting Information). However, high concentration of tryptophan and L‐KYN had inability to defend ferroptosis (Figures S7B–D,S8B, Supporting Information). In contrast, supplementation of 3‐HA at 10–25 µm did not upregulate NRF2 and SLC7A11. To further clarify whether NRF2‐SLC7A11 axis is dispensable for 3‐HA mediated ferroptosis resistance, NRF2 and SLC7A11 KO HT1080 cells were utilized to test the effect of 3‐HA. As shown in Figure S8C,D, Supporting Information, 3‐HA efficiently suppressed ferroptosis in NRF2 KO and SLC7A11 KO cells, suggesting that NRF2‐SLC7A11 axis is dispensable for 3‐HA mediated ferroptosis resistance.

To investigate whether 3‐HA confers cells resistant to ferroptosis as RTA, we performed DPPH and FENIX assays and demonstrated that 3‐HA acts as a potent anti‐oxidant to directly eliminate peroxidized phospholipids (Figure 4F,G). In contrast, 3‐HK exhibited a weak effect to diminish oxidized lipids. KYNU is an enzyme which converts 3‐HK to 3‐HA, whereas HAAO generates quinolinic acid by utilizing 3‐HA as substrate. To validate whether these enzymes regulate 3‐HA mediated ferroptosis, we first examined the expression levels of KYNU and HAAO in a panel of cancer cell lines. HAAO was expressed in almost all cancer cell lines, while KYNU was detectable in CFPAC‐1 and 786‐O cells (Figure 4H). We therefore knocked‐out KYNU in CFPAC‐1 and 786‐O cells to test whether deficiency of KYNU sensitized tumor cells to ferroptosis. As shown in Figure 4I, the expression level of KYNU was completely undetectable in 786‐O KYUN^‐/‐^ clones. Further analysis of LC‐MS based quantitative metabolomics revealed that knockout of KYNU significantly decreased the concentration of cellular 3‐HA, whereas increased the amount of 3‐HK (Figure 4J). As expected, lack of KYNU significantly sensitized cells to ferroptotic cell death (Figure 4K,L). Consistent with this finding, deficiency of KYNU in CFPAC1 cells led to pro‐ferroptosis phenotype (Figure S8E,F, Supporting Information). Furthermore, ectopic expression of KYNU in KYNU‐/‐ cells markedly suppressed the level of ferroptotic cell death (Figure 4M,N). To validate whether KYNU promotes tumor development in vivo, we generated KYNU‐overexpressing B16 cells (Figure S8G, Supporting Information) and performed xenograft assay. As shown in Figure S8H,I, Supporting Information, ectopic expression of KYNU significantly accelerates tumor growth in vivo, indicating that KYNU‐mediated 3‐HA biosynthesis is crucial for ferroptosis escape of tumor cells.

### 2.6 Inactivation of HAAO Accelerates Ferroptosis‐Targeted Tumor Growth

**Figure 5:**
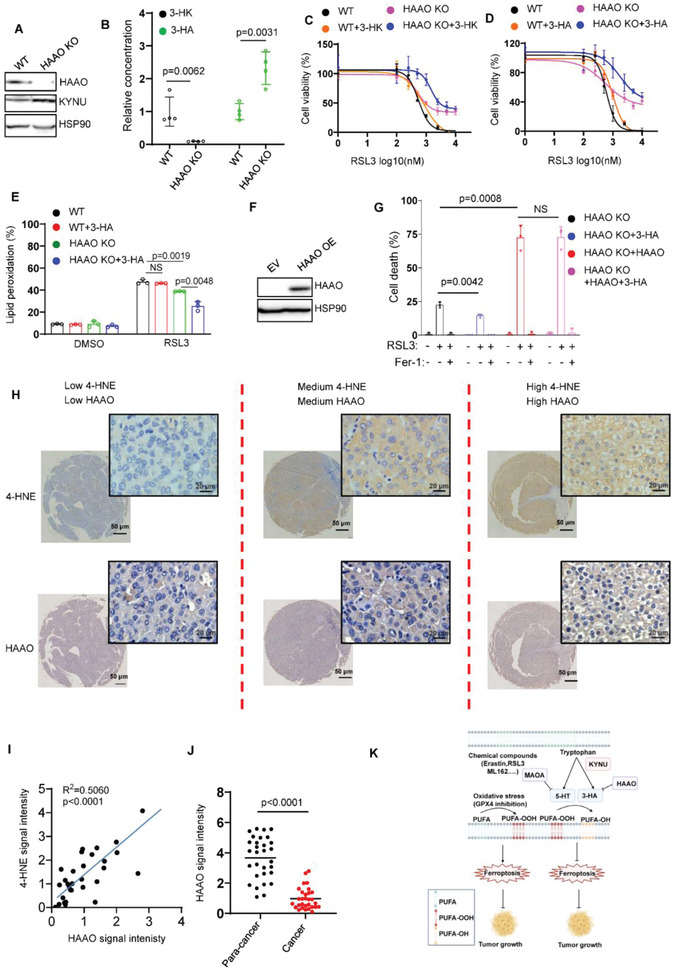
Inactivation of HAAO renders cells resistant to ferroptosis. A) Western blot analysis of HAAO in PANC1 HAAO^‐/‐^ cells. B) LC‐MS based quantitative metabolomics of cellular 3‐HK and 3‐HA in PANC1 WT and HAAO^‐/‐^ cells. C) Dose‐dependent toxicity of RSL3 induced cell death in PANC1 WT and HAAO^‐/‐^ cells with or without 3‐HK treatment (20 µm) for 24 h. D) Dose‐dependent toxicity of RSL3 induced cell death in PANC1 WT and HAAO^‐/‐^ cells with or without 3‐HA treatment (10 µm) for 24 h. E) Lipid peroxidation of PANC1 WT and HAAO^‐/‐^ cells treated with RSL3 and 3‐HA (5 µm). F) Western blot analysis of HAAO in PANC1 HAAO^‐/‐^ cells transfected with empty or HAAO vector. G) PANC1 HAAO^‐/‐^ cells transfected with empty or HAAO vector were treated with RSL3 (2.5 µm) and 3‐HA (5 µm) for 8 h. H) Representative immunohistochemical staining (IHC) of liver cancer samples. I) Dot plot depicting the relevance of the signal intensity of HAAO and 4‐HNE IHC staining in the indicated liver cancer samples. J) HAAO signaling intensity in para‐cancer and liver cancer tissues indicates HAAO is lower expressed in liver cancer tissues. K) Model of tryptophan metabolism‐mediated ferroptosis inhibition.

As HAAO is almost detectable in a panel of cancer cell lines, we guess that degradation of 3‐HA by HAAO might confer cells sensitive to ferroptosis. We therefore generated HAAO KO clones in PANC‐1 cell lines (**Figure** **5**A). We first performed LC‐MS assay to detect the change of 3‐HK and 3‐HA in HAAO KO cells. As expected, deficiency of HAAO markedly increased the abundance of 3‐HA and decreased the accumulation of 3‐HK (Figure 5B). Furthermore, HAAO^‐/‐^ cells showed lower lipid peroxidation and exhibited more robust ferroptosis resistance than WT cells (Figure 5C). Similar phenotype was achieved in CFPAC1 expressing HAAO sgRNA (Figure S8J,K, Supporting Information). The aforementioned data revealed that low concentration of 3‐HK was unable to efficiently protect WT cells against ferroptosis. However, genetic silencing of HAAO remarkedly enhanced 3‐HK mediated ferroptosis inhibition (Figure 5C). Supplementation of low concentration of 3‐HA in HAAO^‐/‐^ cell line renders cells more resistant to ferroptosis, whereas no significant effect in WT cells (Figure 5D,E). Moreover, ectopic expression of HAAO in HAAO‐deficient cells largely abolished the protective role of 3‐HA (Figure 5F,G). Together, these data revealed that HAAO abrogates 3‐HK or 3‐HA mediated ferroptosis resistance to inhibit tumor cell growth via depleting cellular 3‐HA.

Through the gene expression analysis of TIMER database, we found that HAAO expression was lower in multiple cancers including breast cancer, liver cancer et al (Figure S9A, Supporting Information). Therefore, we employed the liver cancer as the model to test whether HAAO is correlated with ferroptosis signature in vivo. As expected, IHC staining of HAAO was highly related to 4‐HNE staining in the human liver cancer tissue microarray (Figure 5H,I). Notably, HAAO signaling intensity is lower in liver cancer than para‐cancer tissues (Figure 5J), indicating HAAO is a potential tumor suppressor. Moreover, Kaplan–Meier survival analysis demonstrated that high expression of HAAO is associated with improved overall survival in patients with other types of cancers including breast, kidney, liver cancer, rectum adenocarcinoma, and others (Figure S9B, Supporting Information). In addition, we analyzed the correlation of HAAO with well‐known ferroptosis‐related genes such as SLC7A11, SLC3A2, ACSL1, and ACSL3 using GEPIA database (http://gepia2.cancer‐pku.cn). The data revealed that the expression level of HAAO in liver cancer is negatively correlated with ferroptosis‐resistant genes including SLC7A11, SLC3A2, and ACSL3, whereas positively correlated with ferroptosis‐sensitive gene ACSL1 (Figure S9C, Supporting Information). Taken together, these data demonstrate that HAAO is positively correlated with ferroptosis responses in tumorigenesis, implying a potential role of HAAO as a tumor suppresser via ferroptosis.

## 3 Discussion

Cysteine‐mediated synthesis of GSH has been long thought as the major pathway to defend ferroptosis among a variety of amino acids. Intracellular cysteine is derived from cystine uptake through system x_c_‐ and de novo biosynthesis of cysteine via transamination pathway are the major source of cysteine. However, whether amino acids‐derived metabolites suppress ferroptosis via cysteine‐independent manner remains largely unknown.

Here we identify and establish an essential role for tryptophan metabolism in ferroptotic cell death. 5‐HT and 3‐HA are the most potent ferroptosis suppressor as RTAs. 5‐HT has been reported to involve in a variety of cellular function via HTRs. However, our data demonstrate that 5‐HT induced ferroptosis resistance is independent of HTRs‐mediated pathway. We also discover that 5‐HT could completely block ferroptotic cell death in most cancer cell lines with no or low expression of MAOA, whereas it has no effect on Be2C cells with high expression of MAOA. Subsequent experiments reveal that genetic inactivation of MAOA efficiently restores accumulation of 5‐HT and rescues 5‐HT mediated ferroptosis inhibition. 3‐HA efficiently accelerates tumor growth in vivo with or without IKE treatment. As KYNU and HAAO are the key genes mediating the levels of 3‐HA, KYNU, or HAAO knockout cell lines are established. Deficiency of KYNU significantly sensitizes cells to ferroptosis, whereas loss of HAAO promotes accumulation of 3‐HA and confers cells resistant to ferroptotic cell death.

In our study, we use RSL3 as a major ferroptosis inducer to validate the protective effect of 5‐HT and 3‐HA. However, we recognize the fact that the direct GPX4 inhibitors such as RSL3 and ML‐162 covalently bind the selenocysteine residue via an activated alkyl chloride.^[^ ^19^ ^]^ Their high chemical reactivity results in a relatively low proteome‐wide selectivity, which limits their use as specific GPX4‐targeted compounds in ferroptosis assay. In contrast, ML210 acts in a more selective manner. Therefore, we have provided multiple, complementary approaches to detect anti‐ferroptosis role of the antioxidants. In addition, we use BODIPY 581/591 to detect general peroxidized lipids in the cells. However, it must be noted that inhibition of BODIPY fluorescence by RTA might not reflect the real ability to diminish lipid peroxidation, since the reaction rate of BODIPY with peroxyl radicals is slower than that of RTA.^[^ ^20^ ^]^ Therefore, it is better complimented with other methods such as MDA, 4‐HNE, or lipidomics to access lipid peroxidation.

Our work demonstrated that supplementation of 3‐HA remarkedly inhibits ferroptosis in HT1080, 786‐O, OVCAR8 cells whereas 3‐HK displays distinct effect depending on cell type. Consistent with the finding of Fiore in Hela cells, supplementation of 3‐HK markedly abrogates ferroptosis in HT1080, OVCAR8 cells. However, weak protective effect was observed in 786‐O cells. Notably, we found that L‐KYN has no effect on RSL3 or erastin‐induced ferroptosis in HT1080, 786‐O, OVCAR8 cells, whereas Fiore et al revealed that L‐KYN efficiently blocked erastin‐induced ferroptosis in Hela cells, indicating that tryptophan metabolites‐mediated ferroptosis inhibition is highly cell‐type specific and depends on many factors such as cell confluence, ferroptosis inducer, nutrient content and dose of compounds. In our study, we used HT1080, 786‐O, OVCAR8 to explore the mechanism of tryptophan metabolites‐regulated ferroptosis. HT1080, 786‐O, OVCAR8 cells are very sensitive to RSL3 induced ferroptosis, suggesting that these cell lines are appropriate models to study RSL3 induced cell death. However, the limitation of 786‐O and OVCAR8 cell lines is that these two cell lines are erastin‐insensitive in our hands and not suitable for estimating erastin‐induced ferroptosis. Furthermore, Hela cell line in our lab is robustly resistant to erastin‐induced ferroptosis compared to Fiore et al. Therefore, future investigation of ferroptosis should seriously consider about these issues and validate the phenomenon using a panel of cell lines or multiple types of ferroptosis inducers to avoid contradictory conclusion. In addition, Fiore et al revealed that knockdown of KYNU decreased the content of 3‐HA and sensitized cells to ferroptosis, whereas overexpressing KYNU enhanced ferroptosis resistance. Consistent with their findings, we also found that loss of KYNU contributes to decreased content of 3‐HA and enhanced ferroptosis sensitivity. Additionally, ectopic expression of KYUN in KYNU KO cells suppressed ferroptotic cell death, indicating that KYNU is essential for 3‐HA biosynthesis to defend ferroptosis.

## 4 Experimental Section

### Cell Culture and Stable Cell Lines

HT1080, 786‐O, CCC‐HPE‐2, U87MG, ASPC‐1, PANC‐1, PC3, MIA‐PACA‐2, U251, B16F10, Be2C, and CFPAC‐1 cell lines were obtained from the Cell Bank of the Chinese Academy of Science (Shanghai, China) and have been proven to be negative for mycoplasma contamination. No cell lines used in this work were listed in the ICLAC database. All cells were cultured in a 37 °C incubator with 5% CO_2_ and maintained in culture medium supplemented with 10% fetal bovine serum (Biological Industries, Israel), 1% Penicillin/Streptomycin solution. All the cell lines were cultured in Dulbecoo's modified Eagle's medium. HT1080 GPX4^‐/‐^ cell line was a gift from Dr. Minghui Gao (Harbin Institute of Technology, China).

### Plasmids

Full length MAOA, KYNU, HAAO were cloned into pcDNA3.1 (Invitrogen) and Plenti4 vectors. For the sgRNAs of MAOA, KYNU, HAAO, these sgRNAs were constructed to plenti‐CRISPR‐V2 vectors.

### Chemicals

Erastin (S7242), RSL3 (S8155), ferrostatin‐1 (S7243), liproxstatin‐1 (S7699), 4‐HPPA (S2995) were pursued from Selleck. SYTOX Green (S34860) and BODIPY‐C11 581/591 (D3861) were pursued from Invitrogen. L‐kynurenine (K8625), 3‐hydroxy‐kynurenine (H1771), 3‐hydroxyanthranilic acid (148776), 4‐HPLA (H3253), 4‐HB (144088), 4‐HBz (H5501), L‐Carnosine (C9625), Ornithine (O6503), phenylpropionic acid (W288918), L‐pipecolate (P2519) were pursued from Sigma‐Aldrich. *α*‐ketoisocaproate (T5071), Agmatine (T4808L), 2‐ketobutyrate (T5060), 3‐hydroxyisobutyric acid (T4947), hippuric acid (T4815), Benzoic acid (T0833), cinnamoic acid (T2740), *β*‐hydroxy‐*β*‐methylbutyrate (T5550), guanidinoacetic acid (T4238), Histamine (T0965), L‐Tryptophan (T0439), 5‐HTP (T1379), 5‐HT (T2209), 5‐HIAA (T4744), melatonin (T1659), quinolinic acid (T4049), and tyrosine (T0493) were pursued from TOPSCIENCE. Trans‐urocanate (I0002) were pursued from J&K Scientific.

### CRISPR‐Cas9 Mediated Gene Silence

The sgRNA sequences were designed from the website (http://crispor.tefor.net/). sgMAOA: GCCATATTCAGTCAAGAGTT, GCCAAACTCTTGACTGAATA; sgKYNU: ATGCGCTGCACTGTGTCAGC, GGTGGCTCTCCACCTAGATG; sgHAAO: TTCGATGTGATAGTCCTTCC, AGAGGTTTGCCAACACCGTG.

### Amino Acids‐Derived Metabolites Screen

HT1080 cells were plated in 96‐well plates at 5000 cells per well and pre‐incubated with indicated amino acids‐derived metabolites. Then 16 h after pre‐incubation of the metabolites, the cells were treated with 100 nm RSL3 for 24 h. The cell viability was measured using CCK8 and data were collected.

### Cell Viability Assay

For cell viability assay, the cells were seeded at 5000 cells per well in 96‐well plates. 16 h after seeding, the cells were treated with indicated concentration of RSL3. 24 h later, the plates were incubated with CCK8 for 1 h and read at 450 nm. The collected values were normalized to blank well. Then the relative cell viability was normalized to the respective DMSO‐treated wells. Graphpad Prism 8 software was used to plot the regression fit curves.

### Cell Death Assay

For cell death assay, cells were seeded in 24‐well plate with 80% confluences and cultured with compounds for indicated time. The SYTOX Green Nucleic Acid dye (Invitrogen) was added into plates for 15 min incubation at 37 °C, and images were captured at least three randomly chosen fields by an inverted fluorescence microscope (Olympus, Japan). Living and dead cells were counted, and cells stained with green were considered as dead cells, and eventually calculated the ratio of dead cells/ living cells + dead cells.

### FACS‐Based Lipid Peroxidation Assay

Cells were harvested and washed with PBS, then resuspended with PBS containing 5 µm C‐11 BODIPY dye (D3861, Thermo Fisher Scientific) and incubated in the tissue culture incubator for 30 min. Cells were then washed twice with PBS followed by resuspending in 200 µL PBS. Lipid ROS levels were analyzed using a Becton Dickinson FACS Calibur machine through the FL1 channel, and the data were analyzed using FlowJo. In each sample, 5000 cells were analyzed.

### Immunofluorescence of BODIPY‐C11 581/591 Staining

BODIPY‐C11 581/591 was used for in situ detection and location of the lipid peroxidation. Oxidation of lipophilic fluorophore resulted in a change of the fluorescence emission peak from ∼590 nm to ∼510 nm. Cells were plated on the slivers in advance, then the cells were incubated in HBSS containing 2 µm of the probe at 37 °C for 15 min after corresponding compounds treatment. Then the cells were washed with HBSS twice and fixed with 4% paraformaldehyde for 15 min. Afterward, the slides were placed on the cover glasses after washing with HBSS twice. The cells were observed using a Zeiss LSM 880 confocal laser scanning microscope. The following excitation and emission settings were used: Excitation wavelength 1: 488 nm, emission filter 1: 500–560; Excitation wavelength 2: 565 nm, emission filter 2: 560–620.

### Transmission Electron Microscopy

HT1080 cells were plated at 5 × 10^6^ cells in 10 cm tissue culture dishes. After 20 h, cells were treated with vehicle (DMSO; 3.5 h), 3‐HA (10 µm; 3.5 h) or 5‐HT (10 µm; 3.5 h) and RSL3 (100 nm; 3.5 h). Cells were fixed with 2.5% glutaraldehyde in PBS for 1 h at room temperature and overnight at 4 °C, then treated with 1% OsO_4_ in PBS for 1 h. Cells were embedded in Epon812, DDSA, DMP‐30, and MNA. Thin sections were cut on a Leica UC7 ultramicrotome, stained with 1% uranyl acetate and 0.4% lead citrate, and examined under a JEM‐1200EX electron microscope. Pictures were taken on an ORCA‐HR digital camera (Hamamatsu) at 5000–50 000× magnification, and measurements were made using the AMT Image Capture Engine.

### FENIX Assay

Egg PC liposomes (extruded to 100 nm, 1 mm), STY‐BODIPY (1 µm), and indicated concentration of tryptophan, 5‐HT, 3‐HK, 3‐HA, lipro‐1 (2 µm), or vehicle (DMSO) were vortexed in PBS (10 mm, pH7.4), then 200 µL aliquots were incubated in a 96‐well plates (polypropylene, Nunc) for 20 min at 37 °C. Thereafter, DTUN (200 mm in EtOH) were added to the aliquots. The plate was vigorously mixed for 5 min and kinetic data of STY‐BODIPY_OX_ was acquired at 488 nm and 518 nm by Mithras LB940 microplate reader (Berthold Technologies).

### DPPH Assay

The 2,2‐diphenyl‐1‐picrylhydrazyl (DPPH) assay was used to measure the lipid‐radical‐scavenging activities of tryptophan metabolites. The stable radical DPPH (MedchemExpress) was dissolved in DMSO to prepare a 0.1 mm solution. Tryptophan derivatives with different concentrations were added to 100 µL of the DPPH solution in a clear bottom 96‐well plate. The plate was incubated at room temperature for 30 min, and the absorbance at 517 nm was recorded. Measurements were performed in triplicate. Ferrostatin‐1 was used as a positive control.

### Western Blotting and Antibodies

Cellular proteins were extracted using BC‐100 lysis buffer supplemented with 1% protease inhibitor cocktail, and protein concentration was determined by a Bradford protein assay kit (Beyotime, China). Total 20 µg proteins were separated by 10% SDS‐polyacrylamide gel, transferred onto nitrocellulose filter membrane (Millipore Corp, Billerica, MA, USA), blocked with 5% fat‐free milk for 1 h at room temperature. The primary antibodies against MAOA (1:1000; 10539‐1‐AP, Proteintech), MAOB (1:1000; 10602‐1‐AP, Proteintech), DDC (1:1000; ab131282, abcam), KYNU (1:1000; 11796‐1‐AP, Proteintech), HAAO (1:1000; 12791‐1‐AP, Proteintech), GPX4 (1:1000; ab125066, abcam), FSP1 (1:1000; 20886‐1‐AP, Proteintech), DHODH (1:1000; 14877‐1‐AP, Proteintech), GCH1 (1:1000; 28501‐1‐AP, Proteintech), SLC7A11 (1:1000; 12691S, CST), p‐AMPK*α* (1:1000; 2535S, CST), p‐mTOR (1:1000; 5536, CST), ACSL4 (1:1000; ab155282, abcam), 4‐HNE (1:1000; ab46545, abcam), NRF2 (1:1000; ab137550, abcam), 5‐HT1A (1:1000; AF0482, Affinity), 5‐HT1B (1:1000; DF3497, Affinity), 5‐HT1D (1:1000; DF2706, Affinity), 5‐HT1F (1:1000; DF3499, Affinity), 5‐HT2A (1:1000; DF8900, Affinity), 5‐HT2B (1:1000; DF3500, Affinity), 5‐HT2C (1:1000; ab133570, abcam), 5‐HT3A (1:1000; DF2707, Affinity), 5‐HT3B (1:1000; DF10195, Affinity), 5‐HT4 (1:1000; DF3503, Affinity), 5‐HT5A (1:1000; AF0631, Affinity), 5‐HT6 (1:1000; DF3505, Affinity), 5‐HT7 (1:1000; ab133570, abcam), Vinculin (1:1000; 66305‐1, Proteintech) and *β*‐actin(1;3000; 20536, Proteintech) were incubated overnight at 4 °C, sequentially the peroxidase affinipure goat anti‐rabbit or mouse IgG(H+L) (Jackson ImmunoResearch Laboratories) and enhanced chemiluminescence solution (GE Healthcare) were used for visualizing protein expression.

### LC/MS Analysis of Phospholipids

All cell samples were homogenized 60 s with 400 µL water. The 10 µL homogenate was mixed with 190 µL water, and then 480 µL extract solution containing internal standard was added. After 60 s vortex, the samples were sonicated for 10 min in ice‐water bath. Then the samples were centrifuged at 3000 rpm for 15 min at 4 °C. 250 µL of supernatant was transferred to a fresh tube. The rest of the sample was added with 250 µL of MTBE, followed with vortex, sonication, and centrifugation, and another 250 µL of supernatant was taken out. This step was repeated twice. And the supernatants were combined and dried in a vacuum concentrator at 37 °C. Then, the dried samples were reconstituted in 100 µL of resuspension buffer (DCM:MeOH:H_2_O = 60:30:4.5) by sonication on ice for 10 min. The constitution was then centrifuged at 12 000 rpm for 15 min at 4 °C, and 30 µL of supernatant was transferred to a fresh glass vial for LC/MS analysis. The quality control (QC) sample was prepared by mixing an equal aliquot of the supernatants from all of the samples.

The UHPLC separation was carried out using an SCIEX ExionLC series UHPLC System. The mobile phase A consisted of 40% water, 60% acetonitrile, and 10 mmol L^−1^ ammonium formate. The mobile phase B consisted of 10% acetonitrile and 90% isopropanol, and 10 mmol L^−1^ ammonium formate. The column temperature was 40 °C. The auto‐sampler temperature was 6 °C, and the injection volume was 2 µL.

AB Sciex QTrap 6500+ mass spectrometer was applied for assay development. Typical ion source parameters were: IonSpray Voltage: +5500/−4500 V, Curtain Gas: 40 psi, Temperature: 350 °C, Ion Source Gas 1:50 psi, Ion Source Gas 2: 50 psi, DP: ±80 V.

Skyline 20.1 Software was employed for the quantification of the target compounds. The absolute content of individuals lipids corresponding to the IS was calculated on the basis of peaks area and actual concentration of the identical lipid class internal standard (IS), and then absolute content was obtained from diverse internal standard (IS) averaged of the identical lipid class.

### LC/MS Analysis of Tryptophan Metabolites

The liquid chromatography with tandem mass spectrometry analyses were performed using a QTRAP 6500+ LC‐MS/MS system (Sciex, Massachusetts, USA) equipped with a Waters Acquity UPLC BEH C18 column (2.1 × 100 mm, 1.7 µm). L‐Kynurenine, 3‐hydroxy‐kynurenine, 3‐hydroxy‐anthranilate, and 5‐hydroxy‐tryptamine were analyzed in positive ion multiple reaction monitoring (MRM) mode. Mobile phase A consisted of water and 0.1% formic acid. Mobile phase B was 100% methanol. Quinolinic acid was analyzed in negative ion multiple reaction monitoring (MRM) mode. Mobile phase A consisted of water and 10 mm ammonium acetate. Mobile phase B was 100% methanol. The analysis was carried out with the following elution gradient: 0–1 min, 95% phase A; 1–10 min, 95–5% phase A; 10–10.5 min, 5% phase A; 10.5–11.5 min, 5–95% phase A; 11.5–12.5 min, 95–5% phase A; and 12.5–13 min, 5–95% phase A. The column temperature was 40 °C. The autosampler temperature was 15 °C, and the injection volume was 1 µL. In this mode, the acquisition software continuously evaluated the full scan mass spectrum. The electrospray‐ionization (ESI) source conditions were set as follows: ion source gas 1, 50 psi; ion source gas 2, 50 psi; capillary temperature, 500 °C; and spray voltage, 5 kV (positive) or −4.5 kV (negative). The area under the curve for the parent ion to daughter ion transition for each compound was integrated and normalized to the relevant internal standard (IS).

### Xenograft Experiment

To explore the role of tryptophan metabolism in ferroptosis‐mediated tumorigenesis in vivo, tumor xenograft models were established and the experimental protocols were practiced complied with the Policy on the Ethical Use and Care of Animals (School of Basic Medical Sciences, Cheeloo College of Medicine, Shandong University).

In B16F10‐tumor bearing xenograft, 2 × 10^5^ B16F10 cells were administered subcutaneously in C57BL/6J mice (Charles River Laboratories, Beijing, China), and then 5‐HT (10 mg kg^−1^), 3‐HA (20 mg kg^−1^), or IKE (40 mg kg^−1^, T5523, TargetMol) were administered intraperitoneally for ten consecutive days. Tumor size was measured every 2–3 day for 4–5 weeks and tumor volume was calculated: volume (cm^3^) = (length × width^2^) ×0.5. All mice were euthanized and tumors were collected, weighted, and embedded in paraffin for further analysis.

### Patient Specimens and Immunohistochemistry

Hepatocellular carcinoma and kidney renal clear cell carcinoma tissue microarrays were purchased from Wuhan Google Biotechnology Co., Ltd. The study complied with the ethical requirements of Shandong University. Immunohistochemistry was carried out to explore the relevance of HAAO and 4‐HNE in cancers. Briefly, sections were soaked in xylene for dewaxing and graded alcohol for rehydrating, followed by incubated with 2% Tween‐20 for 20 min. Antigen retrieval was performed by placing in citrate buffer in water bath for 15 min at 98 °C to shelter endogenous peroxidase activity, and 5% BSA was added to block nonspecific epitopes. Sections were incubated with primary antibodies against MAOA (1:500), HAAO (1:500) and 4‐HNE (1:200) in a humidified chamber at 4 °C overnight. Sections were cultured with biotin‐labeled secondary antibody and then SABC reagent for 30 min according to the manufacturer's instructions. 3′3‐diaminobenzidine and DAPI dye were used for antigen detection. The immunohistochemistry images were photographed by Multispectral fully electric scanning microscopy imaging system (TissueGnostics, Austria), and calculated mean optical density (MOD) values by Image Pro‐plus software.

### Statistics and Reproducibility

Western blot, imaging results were independently repeated at least twice. For H&E and IHC assays, at least four sample sizes was used. All the other experiments were independently repeated at least three times. Statistical analysis was carried out using Microsoft Excel software and GraphPad Prism to assess the differences between experimental groups. Statistical significance was determined by using a two‐tailed, unpaired Student's *t*‐test with a confidence interval (CI) of 95%. The variance was similar between the compared groups. *p* ≤ 0.05 was denoted as statistically significant. Statistical analysis of all survival curves data was performed using log‐Rank (Mantel‐Cox) test. No data was excluded from the study. For in vitro cell‐based experiments, the investigators were not blinded during data acquisition and analysis. The application of treatments and processing procedures made it difficult for blinding but there was no human bias given all the data were collected independently using instrumentation. For the animal experiments the investigators were not blinded to the group allocation. However, at least two observers measured the phenotypes to alleviate human bias in these data.

## Conflict of Interest

The authors declare no conflict of interest.

## Author Contributions

D.L., C.‐h.L., B.H., and X.Z. contributed equally to this work. Conception and experimental design: D.L. and B.C. Methodology and data acquisition: D.L., C.L., B.H., X.Z., and B.C. Analysis and interpretation of data: D.L., C.Y., R.C., B.C. Manuscript writing: B.C. The content is solely the responsibility of the authors.

## Supporting information

Supporting Information

## Data Availability

The data that support the findings of this study are available on request from the corresponding author. The data are not publicly available due to privacy or ethical restrictions.
