# Supplementary material for: Tryptophan Metabolism Acts as a New Anti‐Ferroptotic Pathway to Mediate Tumor Growth
Source: Adv Sci (Weinh). 2023 Jan 10;10(6):2204006. doi: 10.1002/advs.202204006 (PMC9951368; doi:10.1002/advs.202204006)

ADVANCED SCIENCE
Open Access

Supporting Information

for *Adv. Sci.*, DOI 10.1002/advs.202204006

Tryptophan Metabolism Acts as a New Anti-Ferroptotic Pathway to Mediate Tumor Growth

Dong Liu, Chun-hui Liang, Bin Huang, Xiao Zhuang, Weiwei Cui, Li Yang, Yinghong Yang, Yudan Zhang, Xiaolong Fu, Xiaoju Zhang, Lutao Du, Wei Gu, Xiangdong Wang, Chengqian Yin, Renjie Chai* and Bo Chu**

Supplementary Figure legends:

Figure S1. Tryptophan metabolites inhibits ferroptosis.

A-E, Lipid peroxidation of HT1080 cells treated with RSL3 and tryptophan metabolites.

Figure S2. 5-HT and 3-HA inhibits RSL3-induced lipid peroxidation.

A-B, Immunofluorescence staining of BODIPY 581/591 C11 to detect the level of lipid peroxidation in the cells treated with 5-HT (10μM) (A) or 3-HA (10μM) (B).

Figure S3. Tyrosine metabolism pathway has no obvious effect to inhibit ferroptosis.

A, Electron microscopy analysis showed the change of mitochondrial morphology in HT1080 cells treated with RSL3, 5-HT and 3-HA.

B, Schematic diagram of tyrosine metabolism pathway.

C, Dose-dependent toxicity of RSL3 induced cell death in HT1080 cells upon tyrosine metabolites treatment. Tyrosine, 50μM; 4-HPPA, 50μM; 4-HBz, 50μM; 4-HB, 50μM; 4-HPLA, 50μM.

Figure S4. 5-HT is a potent ferroptosis suppressor.

A, Dose-dependent toxicity of RSL3 induced cell death in HT1080 cells upon 5-HT or melatonin treatment for the indicated concentration.

B, Western blot of NRF2 and NRF2 target genes in HT1080 cells supplemented with 5-HT (20μM) treatment.

C, Cell death measurement of HT1080 WT and NRF2 KO cells upon 5-HT (10μM) treatment for 8h.

D, Dose-dependent toxicity of RSL3 induced cell death in HT1080 and 786-O cells upon 5-HTP treatment for the indicated concentration.

E, Dose-dependent toxicity of RSL3 induced cell death in HT1080 and 786-O cells upon 5-HIAA treatment for the indicated concentration.

F, Cell death of HT1080 cells treated with tryptophan (10μM), 5-HT (10μM), 5-HTP (10μM), 5-HAA (10μM) and RSL3 for the indicated concentration.

Figure S5. 5-HT mediated ferroptosis resistance is independent of 5-HT receptor signaling or classical anti-ferroptosis pathway.

A-B, Cell death measurement of 293T arrestin β1-/- (A) cells and arrestin β2-/- (B) cells treated with RSL3 (2.5μM) and Fer-1 (1μM) for 8h.

C, Dose-dependent toxicity of RSL3 induced cell death in HT1080 cells upon NF449 (1μM), PTX (100ng/ml) and 5-HT (10μM) treatment.

D, Western blot analysis of ferroptosis-related genes in HT1080 cells upon 5-HT (20μM) treatment.

E, Cell death measurement of HT1080 FSP1-/- cells treated with RSL3 (200nM), 5-HT (10μM) and Fer-1 (1μM) for 8h.

F, Cell death measurement of HT1080 GCH1-/- cells treated with RSL3 (200nM), 5-HT (10μM) and Fer-1 (1μM) for 8h.

G, Cell death measurement of HT1080 cells expressing DHODH sgRNA treated with RSL3 (200nM), 5-HT (10μM) and Fer-1 (1μM) for 8h.

H, Cell death measurement of HT1080 SLC7A11-/- cells treated with RSL3 (200nM), 5-HT (10μM) and Fer-1 (1μM) for 8h.

Figure S6. MAOA is a potential tumour suppressor.

A, TIMER database analysis revealed that MAOA is down-regulated in multiple cancer samples.

B, Kaplan–Meier survival curves for patients with indicated tumour types containing low or high expression of MAOA mRNA in the TCGA dataset. The P value was calculated using the log-rank Mantel–Cox test.

C, Representative immunohistochemical staining (IHC) of kidney renal clear cell carcinoma samples.

Figure S7. 3-HA remarkedly decreases ferroptotic cell death in a panel of cancer cell lines.

A, Dose-dependent toxicity of RSL3 induced cell death in B16F10 cells upon 3-HA treatment for the indicated concentration.

B, Dose-dependent toxicity of RSL3 or erastin induced cell death in HT1080 cells upon L-KYN, 3-HK and 3-HA treatment for the indicated concentration.

C-D, Dose-dependent toxicity of RSL3 induced cell death in 786-O (C) and OVCAR8 (D) cells upon L-KYN, 3-HK and 3-HA treatment for the indicated concentration.

Figure S8. 3-HA mediated ferroptosis inhibition is independent of NRF2 pathway.

A, Western blot analysis of NRF2 and SLC7A11 in HT1080 cells upon tryptophan, L-KYN, 3-HK and 3-HA treatment for the indicated concentration.

B, Dose-dependent toxicity of RSL3 induced cell death in HT1080 cells upon tryptophan treatment for the indicated concentration.

C-D, Cell death measurement of HT1080 NRF2-/- (C) and SLC7A11-/- (D)cells treated with RSL3 (200nM), 5-HT (10μM) and Fer-1 (1μM) for 8h.

E, Western blot analysis of KYNU in CFPAC1 KYNU-/- clones.

F, Cell viability of CFPAC1 WT and KYNU-/- cells treated with RSL3 for the indicated concentration.

G, Western blot analysis of KYNU in WT and KYNU-overexpressing B16F10.

H-I. Tumour volumes (H) and weight (I) of WT and KYNU-overexpressing B16F10 xenograft in C57BL/6 mice (n=6).

J, Western blot analysis of HAAO in CFPAC1 cells expressing HAAO sgRNA.

K, Cell death measurement of CFPAC1 cells expressing HAAO sgRNA treated with RSL3 for 8h.

Figure S9. HAAO is a potential tumour suppressor.

A, TIMER database analysis revealed that HAAO is down-regulated in multiple cancer samples.

B, Kaplan–Meier survival curves for patients with indicated tumour types containing low or high expression of HAAO mRNA in the TCGA dataset. The P value was calculated using the log-rank Mantel–Cox test.

C, The correlation of HAAO with ferroptosis-relative genes.

Figure S1

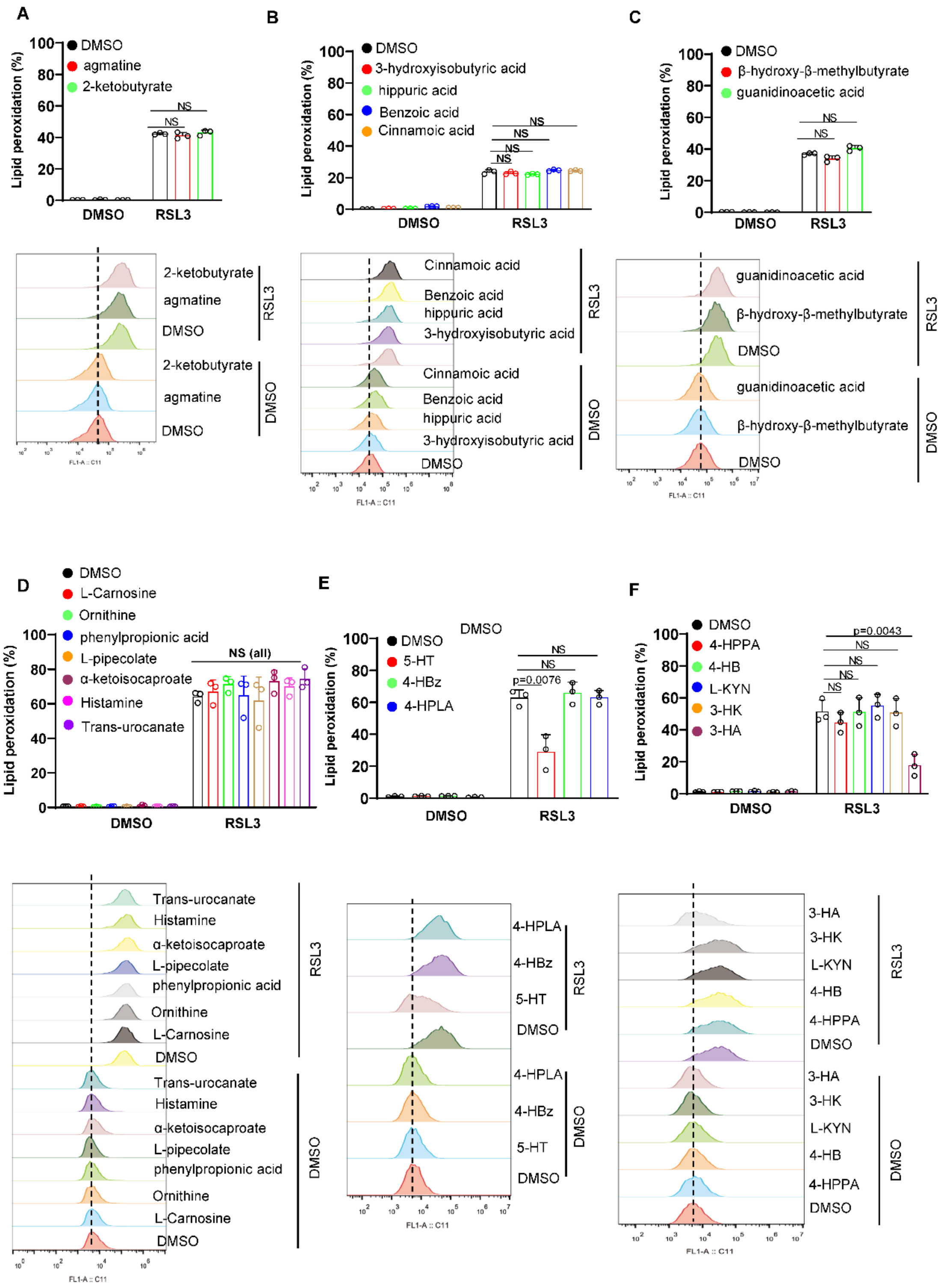
A
B
C
D
E
F
Lipid peroxidation (%)
DMSO
agmatine
2-ketobutyrate
3-hydroxyisobutyric acid
hippuric acid
Benzoic acid
Cinnamoic acid
β-hydroxy-β-methylbutyrate
guanidinoacetic acid
L-Carnosine
Ornithine
phenylpropionic acid
L-pipecolate
α-ketoisocaproate
Histamine
Trans-urocanate
5-HT
4-HBz
4-HPLA
4-HPPA
4-HB
L-KYN
3-HK
3-HA
NS
NS (all)
p=0.0076
p=0.0043
RSL3
FL1-A :: C11

Figure S2

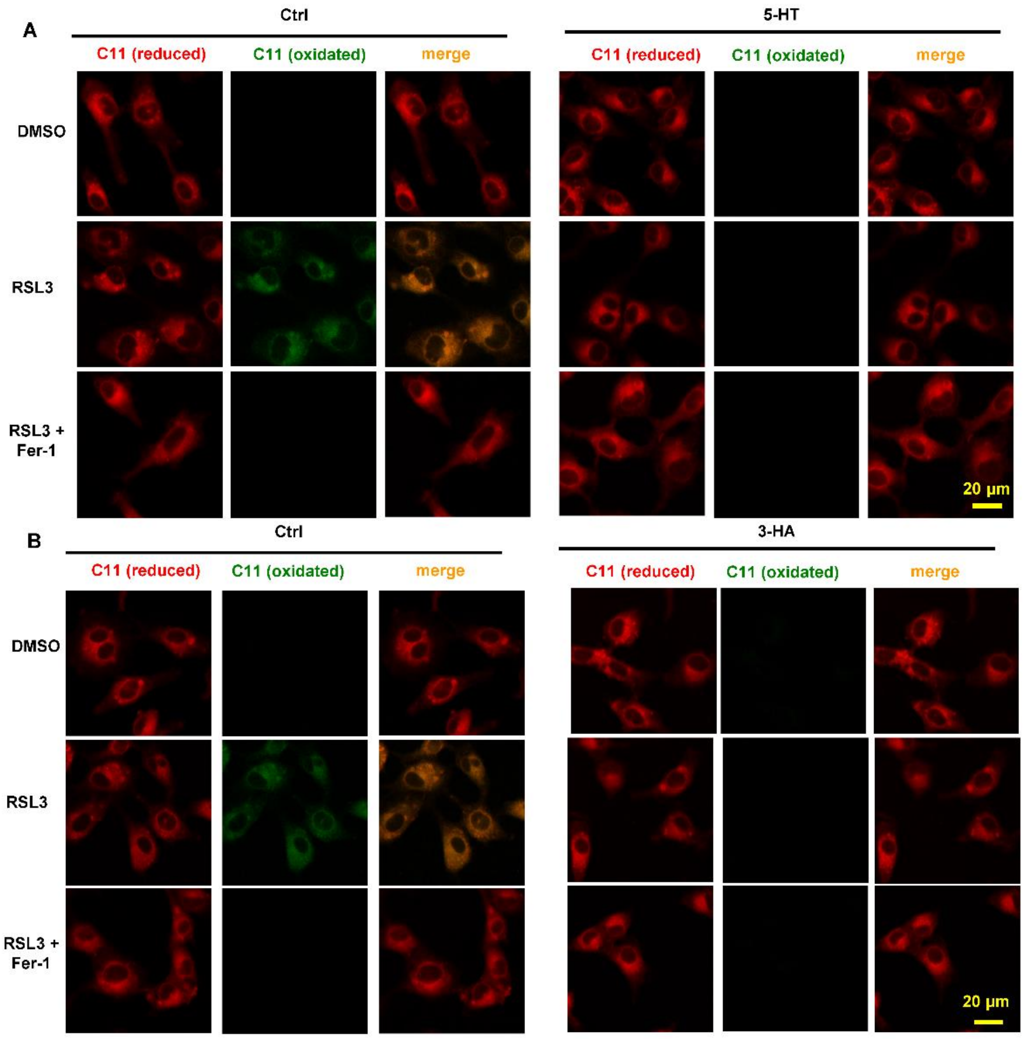
A
Ctrl
5-HT
C11 (reduced)
C11 (oxidated)
merge
DMSO
RSL3
RSL3 +
Fer-1
20 μm
B
Ctrl
3-HA
C11 (reduced)
C11 (oxidated)
merge
DMSO
RSL3
RSL3 +
Fer-1
20 μm

Figure S3

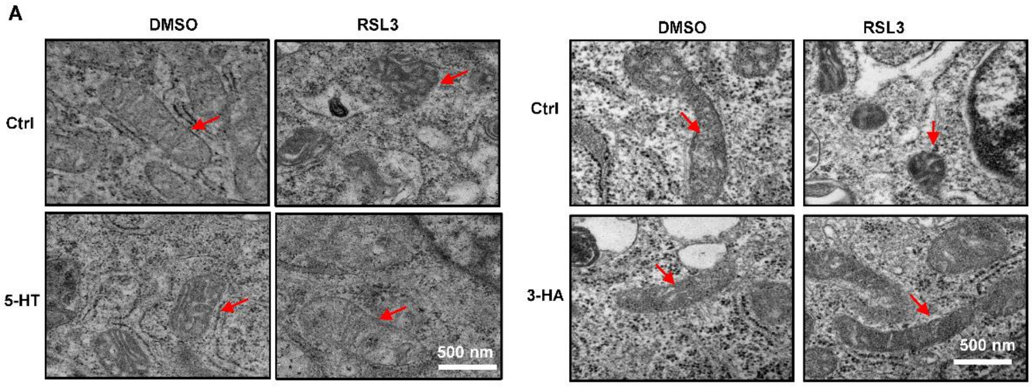
A
DMSO
RSL3
Ctrl
5-HT
500 nm
DMSO
RSL3
Ctrl
3-HA
500 nm

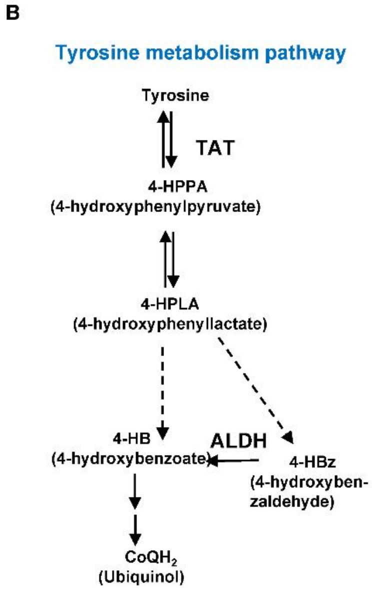
B
Tyrosine metabolism pathway
Tyrosine
TAT
4-HPPA
(4-hydroxyphenylpyruvate)
4-HPLA
(4-hydroxyphenyllactate)
4-HB
(4-hydroxybenzoate)
ALDH
4-HBz
(4-hydroxyben-
zaldehyde)
CoQH2
(Ubiquinol)

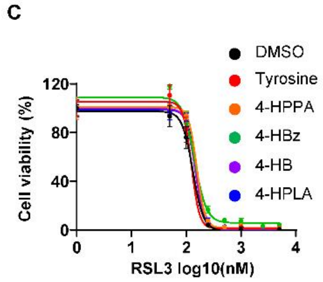
C
DMSO
Tyrosine
4-HPPA
4-HBz
4-HB
4-HPLA
Cell viability (%)
120
80
40
0
0
1
2
3
4
RSL3 log10(nM)

Figure S4

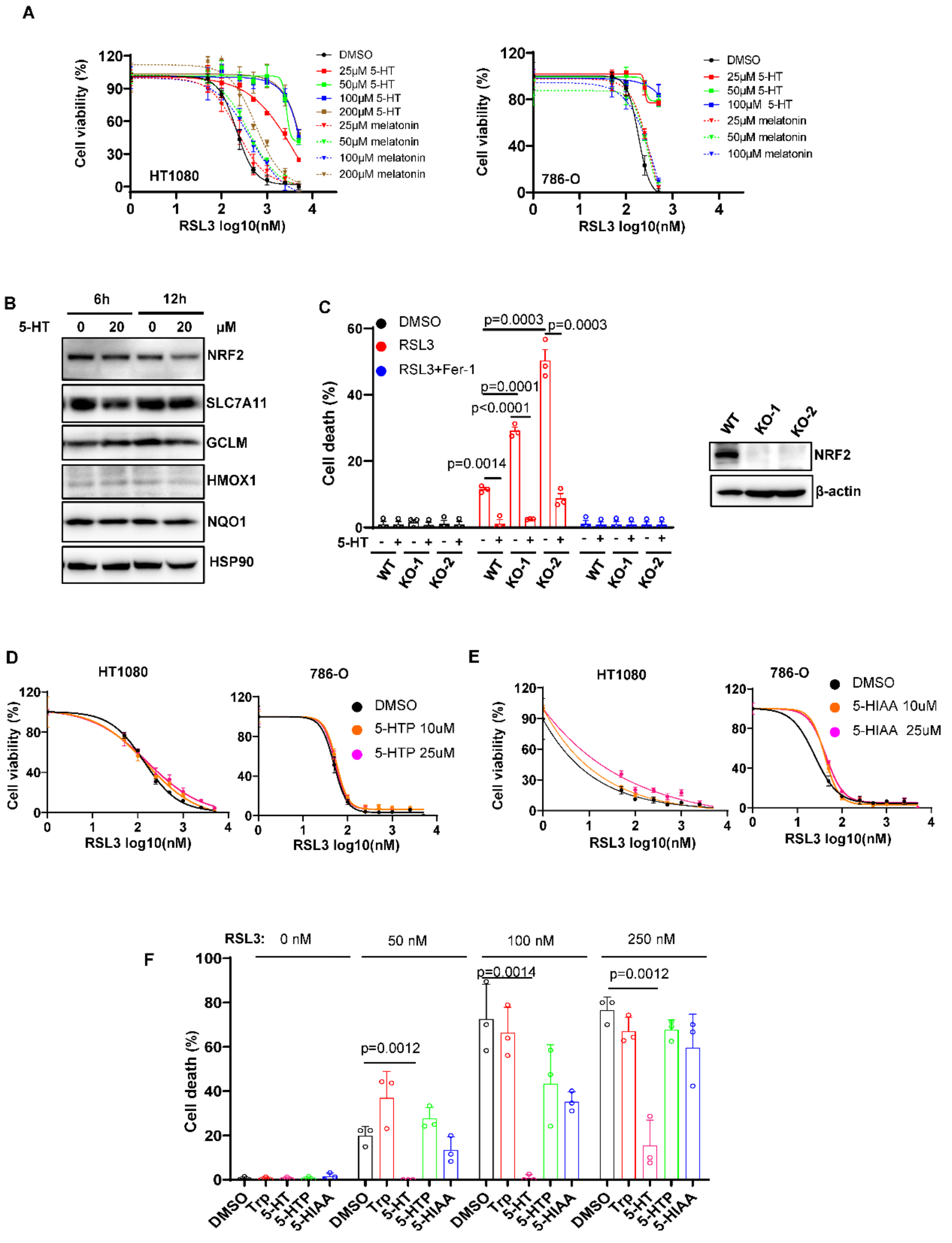
A
Cell viability (%)
HT1080
RSL3 log10(nM)
DMSO
25µM 5-HT
50µM 5-HT
100µM 5-HT
200µM 5-HT
25µM melatonin
50µM melatonin
100µM melatonin
200µM melatonin
786-O
B
6h
12h
5-HT
0
20
µM
NRF2
SLC7A11
GCLM
HMOX1
NQO1
HSP90
C
Cell death (%)
DMSO
RSL3
RSL3+Fer-1
p=0.0003
p=0.0003
p=0.0001
p<0.0001
p=0.0014
5-HT
WT
KO-1
KO-2
β-actin
D
5-HTP 10uM
5-HTP 25uM
E
5-HIAA 10uM
5-HIAA 25uM
F
RSL3:
0 nM
50 nM
100 nM
250 nM
p=0.0012
p=0.0014
p=0.0012
Trp
5-HT
5-HTP
5-HIAA

Figure S5

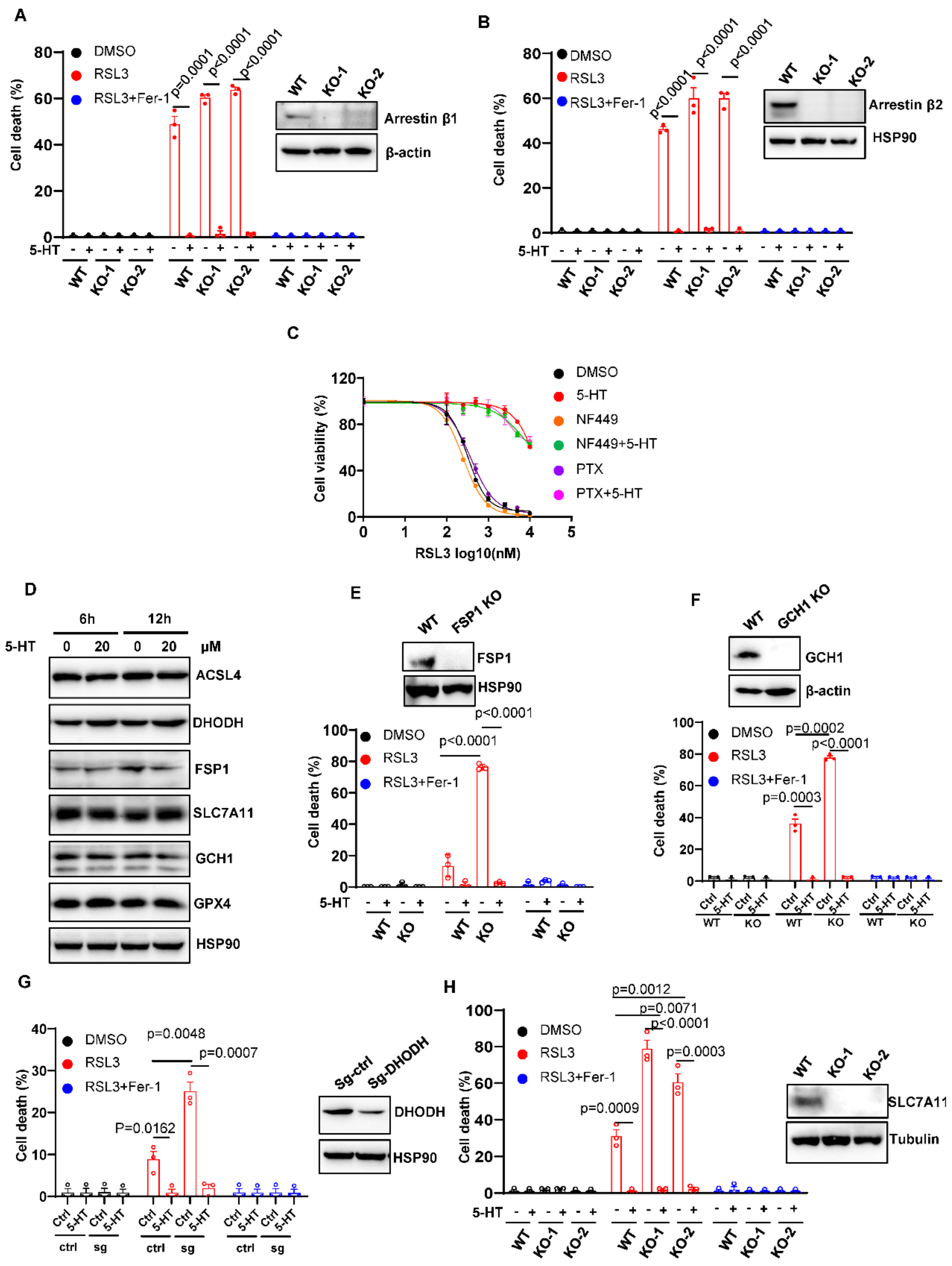
A
DMSO
RSL3
RSL3+Fer-1
Cell death (%)
p<0.0001
p<0.0001
p<0.0001
5-HT
WT
KO-1
KO-2
Arrestin β1
β-actin
B
DMSO
RSL3
RSL3+Fer-1
Cell death (%)
p<0.0001
p<0.0001
p<0.0001
Arrestin β2
HSP90
C
DMSO
5-HT
NF449
NF449+5-HT
PTX
PTX+5-HT
Cell viability (%)
RSL3 log10(nM)
D
6h
12h
5-HT
μM
ACSL4
DHODH
FSP1
SLC7A11
GCH1
GPX4
HSP90
E
WT
FSP1 KO
FSP1
HSP90
p<0.0001
p<0.0001
F
WT
GCH1 KO
GCH1
β-actin
p=0.0002
p<0.0001
p=0.0003
G
p=0.0048
p=0.0007
P=0.0162
Sg-ctrl
Sg-DHODH
DHODH
HSP90
H
p=0.0012
p=0.0071
p<0.0001
p=0.0003
p=0.0009
SLC7A11
Tubulin

Figure S6

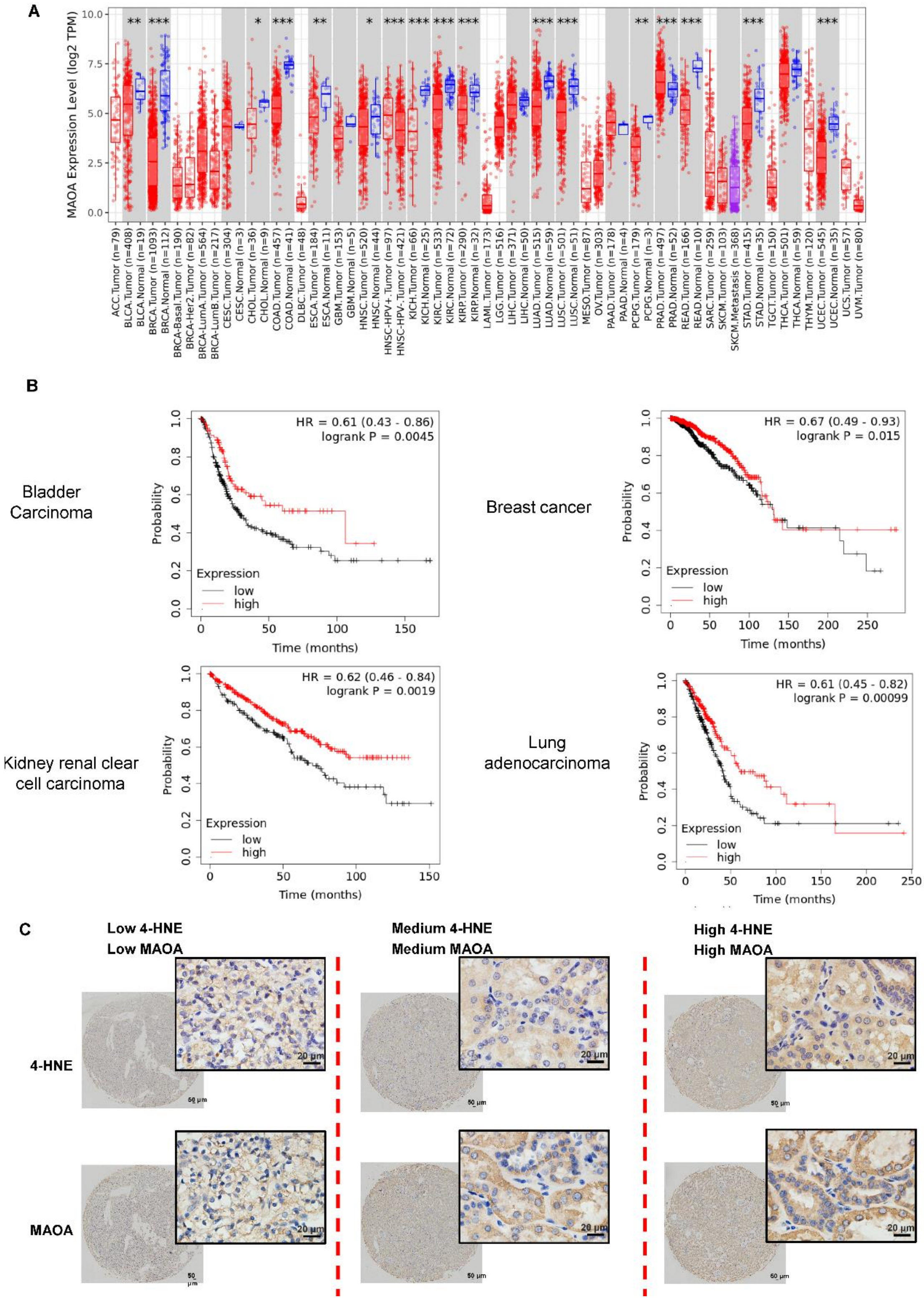
A
MAOA Expression Level (log2 TPM)
ACC.Tumor (n=79)
BLCA.Tumor (n=408)
BLCA.Normal (n=19)
BRCA.Tumor (n=1093)
BRCA.Normal (n=112)
BRCA-Basal.Tumor (n=190)
BRCA-Her2.Tumor (n=82)
BRCA-LumA.Tumor (n=564)
BRCA-LumB.Tumor (n=217)
CESC.Tumor (n=304)
CESC.Normal (n=3)
CHOL.Tumor (n=36)
CHOL.Normal (n=9)
COAD.Tumor (n=457)
COAD.Normal (n=41)
DLBC.Tumor (n=48)
ESCA.Tumor (n=184)
ESCA.Normal (n=11)
GBM.Tumor (n=153)
GBM.Normal (n=5)
HNSC.Tumor (n=520)
HNSC.Normal (n=44)
HNSC-HPV+.Tumor (n=97)
HNSC-HPV-.Tumor (n=421)
KICH.Tumor (n=66)
KICH.Normal (n=25)
KIRC.Tumor (n=533)
KIRC.Normal (n=72)
KIRP.Tumor (n=290)
KIRP.Normal (n=32)
LAML.Tumor (n=173)
LGG.Tumor (n=516)
LIHC.Tumor (n=371)
LIHC.Normal (n=50)
LUAD.Tumor (n=515)
LUAD.Normal (n=59)
LUSC.Tumor (n=501)
LUSC.Normal (n=51)
MESO.Tumor (n=87)
OV.Tumor (n=303)
PAAD.Tumor (n=178)
PAAD.Normal (n=4)
PCPG.Tumor (n=179)
PCPG.Normal (n=3)
PRAD.Tumor (n=497)
PRAD.Normal (n=52)
READ.Tumor (n=166)
READ.Normal (n=10)
SARC.Tumor (n=259)
SKCM.Tumor (n=103)
SKCM.Metastasis (n=368)
STAD.Tumor (n=415)
STAD.Normal (n=35)
TGCT.Tumor (n=150)
THCA.Tumor (n=501)
THCA.Normal (n=59)
THYM.Tumor (n=120)
UCEC.Tumor (n=545)
UCEC.Normal (n=35)
UCS.Tumor (n=57)
UVM.Tumor (n=80)
B
Bladder Carcinoma
HR = 0.61 (0.43 - 0.86)
logrank P = 0.0045
Breast cancer
HR = 0.67 (0.49 - 0.93)
logrank P = 0.015
Kidney renal clear cell carcinoma
HR = 0.62 (0.46 - 0.84)
logrank P = 0.0019
Lung adenocarcinoma
HR = 0.61 (0.45 - 0.82)
logrank P = 0.00099
Probability
Time (months)
Expression
low
high
C
Low 4-HNE
Low MAOA
Medium 4-HNE
Medium MAOA
High 4-HNE
High MAOA
4-HNE
MAOA
20 μm
50 μm

Figure S7

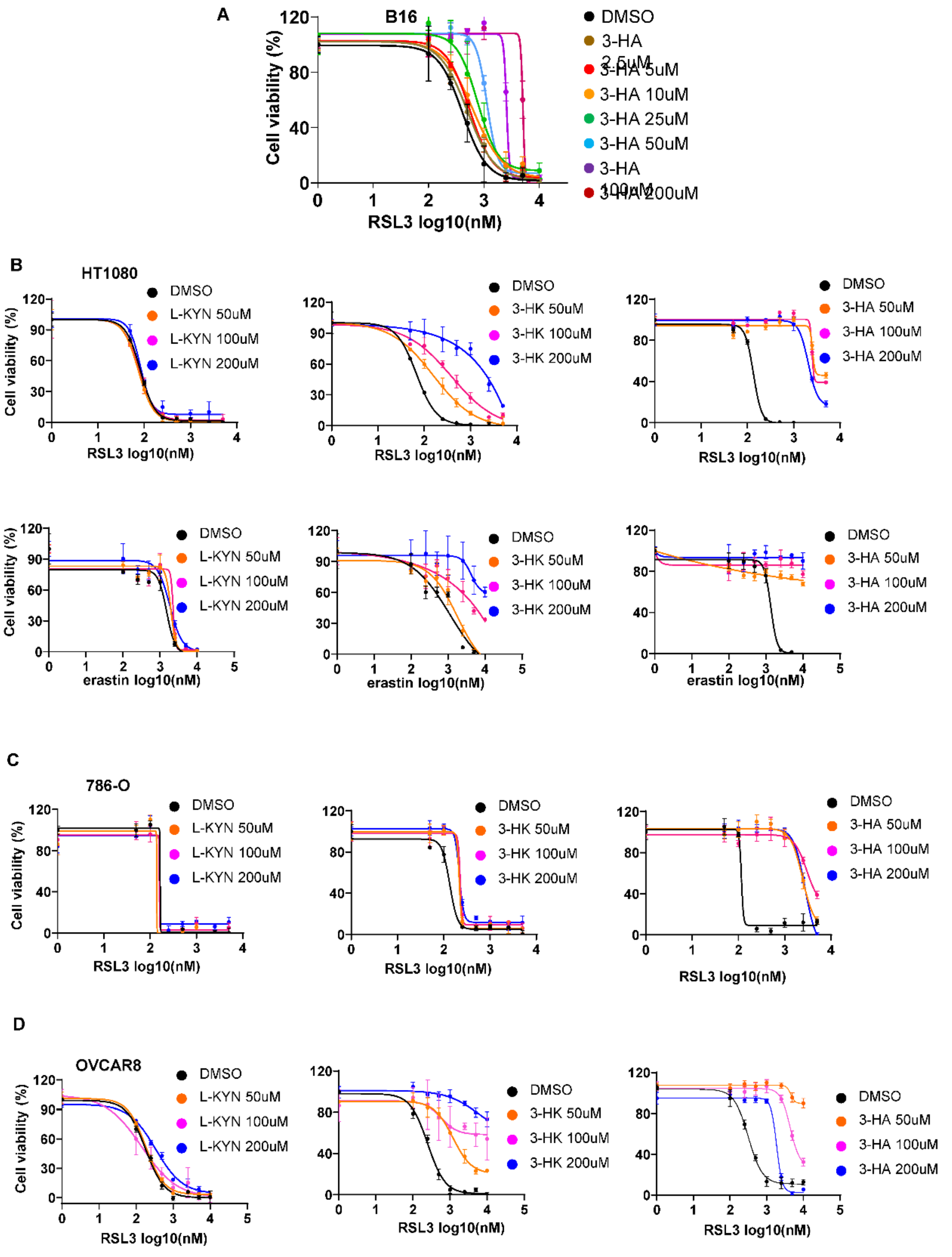
A
B16
Cell viability (%)
RSL3 log10(nM)
DMSO
3-HA 2.5uM
3-HA 5uM
3-HA 10uM
3-HA 25uM
3-HA 50uM
3-HA 100uM
3-HA 200uM
B
HT1080
DMSO
L-KYN 50uM
L-KYN 100uM
L-KYN 200uM
3-HK 50uM
3-HK 100uM
3-HK 200uM
3-HA 50uM
3-HA 100uM
3-HA 200uM
erastin log10(nM)
C
786-O
D
OVCAR8

Figure S8

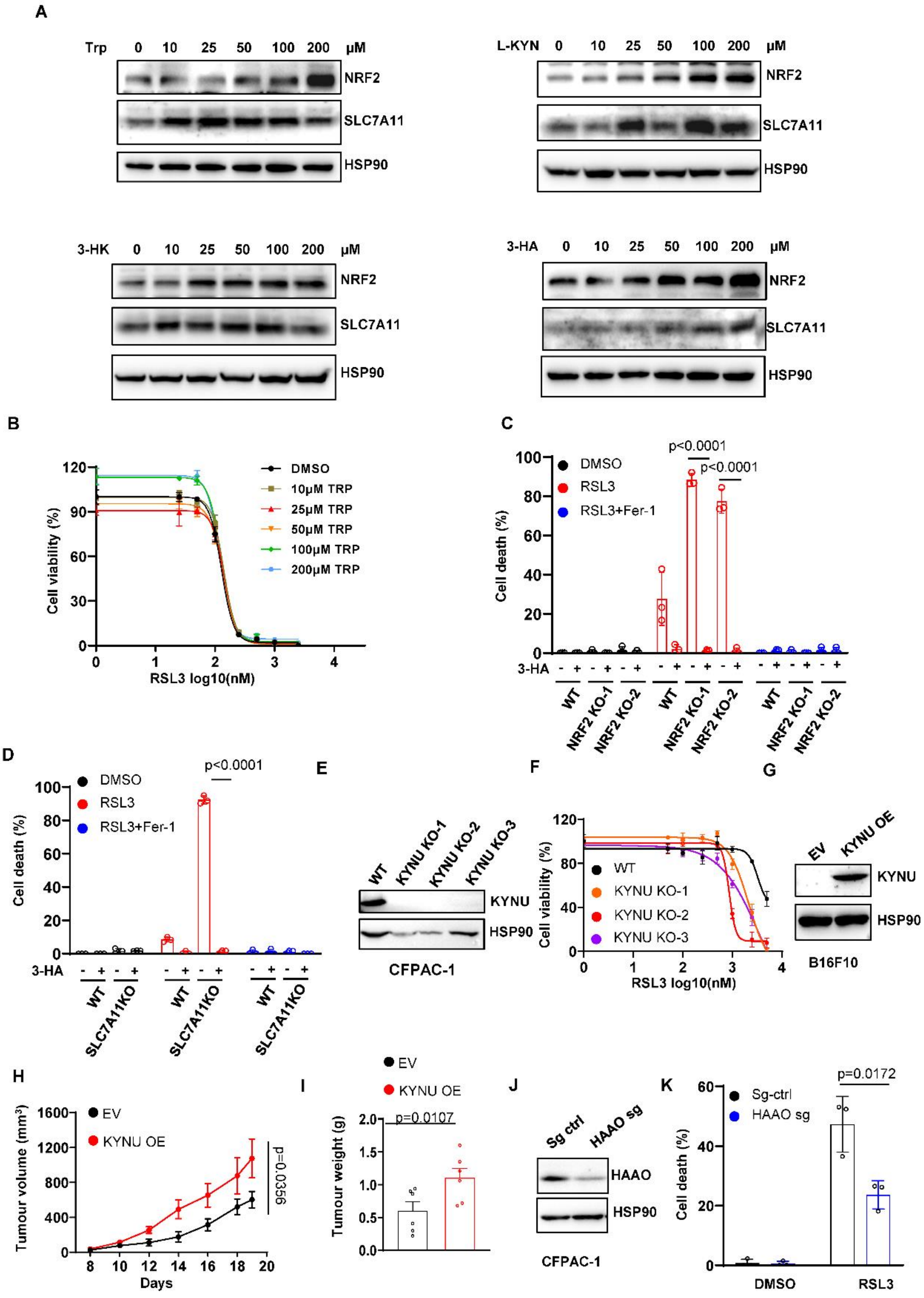
A
Trp 0 10 25 50 100 200 μM
NRF2
SLC7A11
HSP90
L-KYN 0 10 25 50 100 200 μM
3-HK 0 10 25 50 100 200 μM
3-HA 0 10 25 50 100 200 μM
B
Cell viability (%)
RSL3 log10(nM)
DMSO
10μM TRP
25μM TRP
50μM TRP
100μM TRP
200μM TRP
C
Cell death (%)
DMSO
RSL3
RSL3+Fer-1
p<0.0001
3-HA
WT
NRF2 KO-1
NRF2 KO-2
D
SLC7A11KO
E
KYNU KO-1
KYNU KO-2
KYNU KO-3
KYNU
CFPAC-1
F
KYNU KO-1
KYNU KO-2
KYNU KO-3
G
EV
KYNU OE
B16F10
H
Tumour volume (mm³)
Days
p=0.0356
I
Tumour weight (g)
p=0.0107
J
Sg ctrl
HAAO sg
HAAO
K
Sg-ctrl
p=0.0172

Figure S9

A

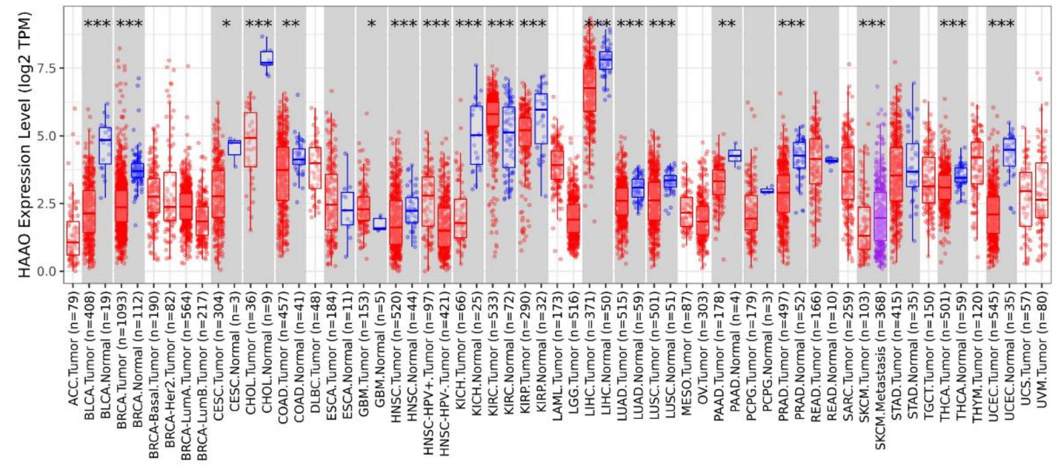

B

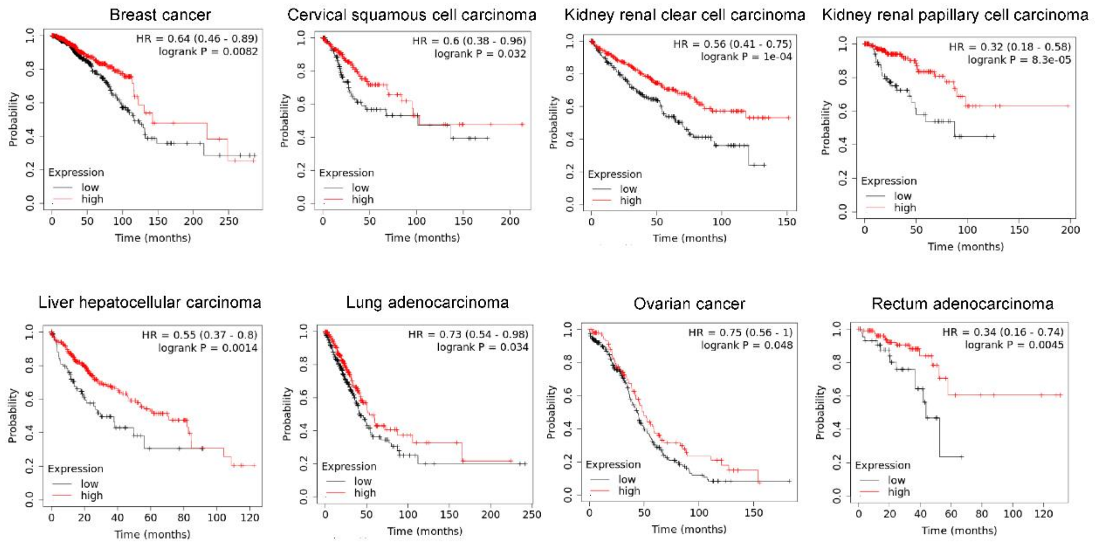

C

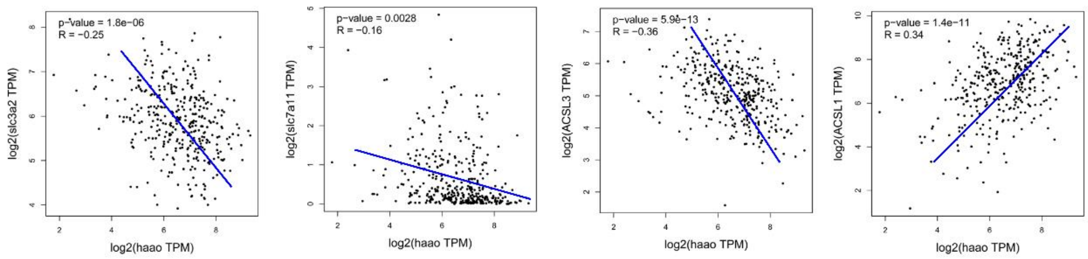

Supplement: Supplementary file 1 — Supporting Information [file ADVS-10-2204006-s001.pdf]
